# Advancing Scoliosis Treatment with Patient-Specific Functionally Graded NiTi-SMA Rods: Key Considerations and Development Objectives

**DOI:** 10.3390/bioengineering13020216

**Published:** 2026-02-13

**Authors:** Shiva Mohajerani, Alireza Behvar, Athena Jalalian, Ahu Celebi, Mohammad Elahinia

**Affiliations:** 1 Mechanical, Industrial and Manufacturing Department, The University of Toledo, 2801 Bancroft St., Toledo, OH 43606, USA; alireza.behvar@rockets.utoledo.edu (A.B.); mohammad.elahinia@utoledo.edu (M.E.); 2 Department of Design, Production, and Management, University of Twente, Drienerlolaan 5, 7522 NB Enschede, The Netherlands; a.jalalian@utwente.nl

**Keywords:** functionally graded structures, shape memory alloys, additive manufacturing, scoliosis surgical treatment, patient-specific instrumentation, NiTi rods

## Abstract

This review develops a materials-to-clinic framework for patient-specific, functionally graded (FG) NiTi shape memory alloy (SMA) rods as a complementary paradigm for scoliosis correction that targets durable alignment with motion preservation. The article synthesizes the thermomechanical basis of NiTi (thermoelastic martensitic transformation, near constant superelastic plateau, and hysteretic damping) while leveraging additive manufacturing (AM) capabilities to spatially program transformation temperatures (e.g., A_f_), effective stiffness, and geometric inertia along the rod. Consolidated process–structure–property linkages are provided for the PBF-LB, DED, and BJAM routes, together with contamination and composition-control strategies (mitigation of Ni volatilization; management of O/C uptake; gradient heat treatments) and segment-level quality assurance (DSC mapping, micro-CT, EBSD/indentation, and bench bending/torsion in physiologic media). Building on clinical curve classification, the methodology formalizes a grading mask and target moment vector that drive multi-objective optimization of the segmental A_f_, relative density/architecture, and cross-section, followed by route-specific build plans and acceptance tolerances. A phenomenological constitutive description provides the forward map from local design variables to temperature-dependent moment–curvature loops for finite element verification and uncertainty control. Surgical handling and activation policies are codified (cold shaping in martensite and controlled intra-/postoperative warming within tissue-safe bounds), and a translational roadmap is outlined, encompassing prospective calibration of classification-to-design mappings, AM process maps with in situ monitoring, digital twin planning, and long-horizon fatigue/corrosion protocols. The proposed graded structures provide an adaptive transformation temperature gradient and tunable mechanical response, representing an important design direction toward 3D-printed, patient-specific SMA rods for durable, adjustable, and efficient scoliosis correction.

## 1. Introduction

**Figure 1:**
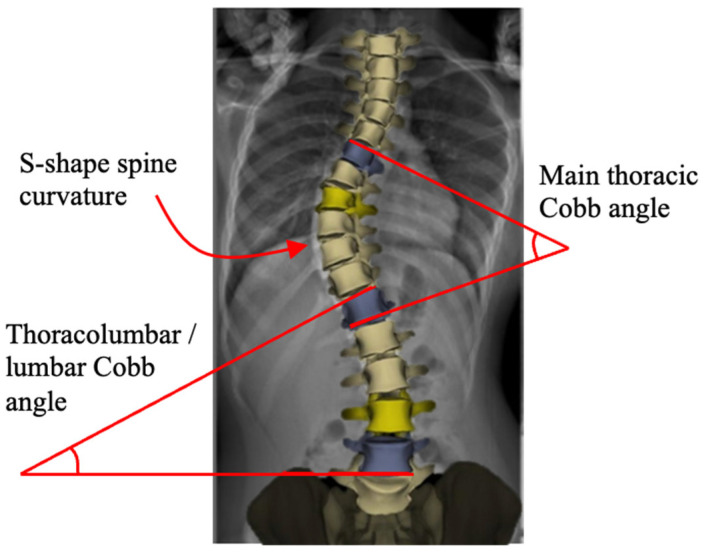
S-shaped scoliotic spine. Cobb angles are determined on the thoracic and lumbar spine regions. The X-rays and patients’ photos are from the patient group recruited for a project which had been approved by the domain specific review board (DSRB) and ethics committee at National University Hospital in Singapore. All patients involved in the study had been properly consulted, and their approval and informed consents were obtained.

Scoliosis is a three-dimensional deformity of the spine causing S- and/or C-shape spine curvature in the coronal plane (see Figure 1) [1,2]. It is clinically characterized by the Cobb angle [3], which quantifies the level of the bending disorders of the spinal column. Scoliosis is a significant progressive musculoskeletal disorder with both physical and psychosocial implications. As scoliosis progresses, it can lead to pain, cosmetic deformity, respiratory dysfunction, and reduced quality of life. The burden of disease includes not only medical complications but also impacts on mental health, function, and long-term care [4,5]. The most common form, adolescent idiopathic scoliosis (AIS), makes up 80–85% of cases and affects between 1% and 4% of the population [6]. Management strategies vary with patient age, curve severity, and skeletal maturity [7]. Mild cases are typically managed conservatively through observation or bracing, while moderate to severe curves often require surgical correction [8]. However, the existing surgical techniques present substantial trade-offs between factors such as correction level, spinal growth, motion preservation, and stability.

Posterior spinal fusion (PSF) is widely regarded as the gold standard for correcting structural scoliosis, especially AIS, because it provides durable deformity correction, halts curve progression, and stabilizes the spine in the long term. In PSF, instrumentation (typically pedicle screws and rods) is used to realign the spine, and bone grafts facilitate permanent arthrodesis of the involved vertebral segments. PSF achieves high coronal curve correction and a low rate of unplanned revisions [9,10]. From a biomechanical perspective, while fusion achieves robust structural stabilization, it eliminates motion within the fused segments [11,12]. This rigid fixation can lead to altered load distribution across adjacent unfused segments, potentially accelerating degenerative changes in intervertebral discs and facets (adjacent segment degeneration). Furthermore, because PSF locks spinal segments in a fixed position, it does not accommodate ongoing vertebral growth or spinal remodeling and, thus, cannot adapt after initial correction, raising concerns about long-term alignment stability, particularly in growing adolescents. In younger patients, definitive fusion at an early age can severely restrict spinal and thoracic growth, potentially compromising pulmonary development and overall trunk height [13]. Non-fusion instrumentation techniques are an effective alternative way to address these problems and are gaining popularity for treating AIS.

**Figure 2:**
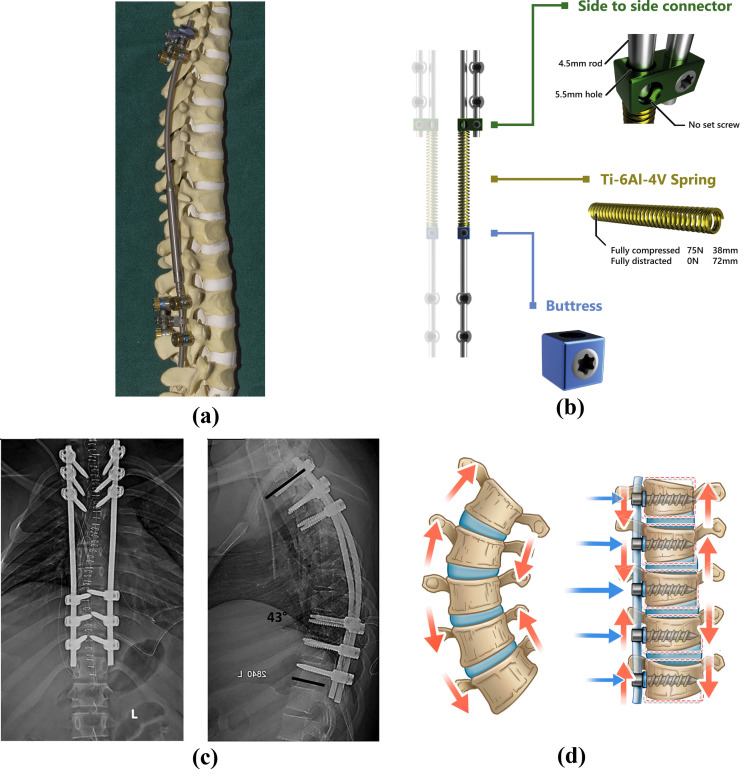
Representation of currently available techniques for scoliosis surgery: (**a**) magnetically controlled growing rod fixed to a spine mode [39], (**b**) spring distraction system concept [24], (**c**) posterior spinal instrumented fusion [40], (**d**) vertebral body tethering [41].

Non-fusion instrumentation techniques aim to mitigate curve progression or correct spinal deformity while allowing for spinal growth, preserving motion, and delaying eventual spinal fusion [14,15]. However, these techniques, while offering better flexibility and a faster recovery, have several disadvantages, including a higher reoperation rate, potentially less effective correction for severe curves, unknown long-term outcomes, and mechanical risks like implant failure or breakage [16,17]. The most common non-fusion technique is vertebral body tethering (VBT), also known as the “tether” or Anterior Scoliosis Correction. This technique uses an anterior or lateral approach to place screws in vertebral bodies on the convex side of the curvature and connect them with a flexible tether. Tether tensioning applies asymmetric compression, theoretically slowing growth on one side of the spine while allowing for continued growth on the opposite side, thus gradually correcting curvature. In principle, VBT preserves segmental spinal motion and allows for continued spinal growth [17]. Initial clinical reports indicate that VBT can achieve substantial curve correction while maintaining motion in appropriately selected patients, but outcomes have been inconsistent, and long-term data are still emerging (Figure 2). Thus, careful patient selection (e.g., moderate, flexible curves in skeletally immature patients) is critical to tethering result optimization [18].

Magnetically controlled growth rods (MCGRs) have emerged as a transformative innovation in the management of scoliosis, particularly early-onset scoliosis. They provide a non-fusion alternative that integrates mechanical precision with minimally invasive control [19]. The system operates through an internal telescopic rod containing a rare-earth magnetic actuator that can be remotely adjusted by an external magnetic field, enabling gradual spinal distraction without repeated surgical intervention. Clinically, MCGRs have demonstrated substantial efficacy in maintaining spinal alignment, promoting thoracic growth, and reducing the cumulative surgical burden associated with traditional growing rod systems [20]. However, device-related challenges such as loss of distraction, mechanical wear, metallosis, and actuator stalling have been observed, highlighting the importance of rigorous quality control and design optimization [21,22]. The safety profile of MCGRs remains favorable when employed under appropriate clinical protocols, yet long-term mechanical integrity and tissue–device interactions warrant further investigation [23]. Optimal patient selection is critical to clinical success, typically encompassing skeletally immature patients with progressive spinal deformities exceeding 40° and adequate spinal flexibility.

Spring-distraction-aided growth guidance systems represent an evolution in non-fusion scoliosis correction by combining continuous, low-magnitude distraction forces with physiological spinal growth [24]. Unlike traditional growing rods that rely on periodic lengthening, this approach employs an internal spring mechanism that provides dynamic, self-adjusting tension along the spinal axis, thereby maintaining corrective forces throughout the growth phase. From a biomechanical standpoint, the system promotes gradual vertebral remodeling under quasi-static loading, which more closely mimics natural growth stimuli and reduces stress shielding effects commonly observed in rigid instrumentation. Clinically, spring-based systems have demonstrated improved spinal alignment maintenance and reduced complication rates associated with repeated surgical interventions [25,26]. The continuous distraction mechanism minimizes abrupt force changes, decreasing the likelihood of implant loosening, junctional kyphosis, and soft tissue irritation. However, potential concerns persist regarding long-term fatigue resistance of the spring element, mechanical hysteresis, and the risk of over-distraction in cases of asymmetric growth or insufficient curve flexibility. Therefore, rigorous preoperative assessment, including curve magnitude, flexibility index, and patient growth potential, is essential for appropriate candidate selection. Ideal patients are skeletally immature individuals with progressive yet flexible deformities and without severe vertebral malformations. From an analytical perspective, the integration of real-time biomechanical monitoring and finite element modeling can further optimize spring stiffness, preload calibration, and stress distribution to achieve balanced correction while maintaining physiological growth dynamics.

The fusion and non-fusion interventions embody a fundamental trade-off: fusion provides reliable, immediate correction but at the cost of mobility and potential long-term degeneration, whereas non-fusion strategies preserve the capacity of the spine to grow but carry uncertainty regarding durability, corrective magnitude, and adaptability [27]. A recent meta-analysis comparing PSF and VBT found that PSF resulted in a significantly greater percentage of coronal curve correction postoperatively and at two years, with lower odds of unplanned surgical revisions [28,29]. However, PSF is associated with longer operative times, greater intraoperative blood loss, more extensive spinal instrumentation, and potential long-term sequelae, such as adjacent segment degeneration; loss of spinal mobility; and, in pediatric patients, compromised growth. Early studies suggest that VBT can result in substantial curve correction, with the potential to avoid spinal fusion in many cases [17,30,31]. VBT has been associated with reduced surgical blood loss, shorter hospitalization, quicker return to activity, and better segmental mobility compared to PSF. However, VBT is also linked to high rates of tether breakage (reported in 13–50% of patients at three years) and subsequent loss of correction or need for revision surgery. Long-term data on outcomes, disc health, over-correction risks, and biomechanical consequences of tether failure remain limited. The consequences of disc and facet joint compression over time, and the effects of tether breakage on spinal dynamics, are still under investigation. Indeed, expert surveys indicate that significant equipoise remains among surgeons regarding the choice between fusion and tethering, underscoring the lack of consensus on an optimal treatment paradigm in AIS. The limitations intrinsic to both fusion and non-fusion approaches highlight an unmet need for scoliosis implants that can deliver controlled, gradual corrective forces; adapt to spinal growth and remodeling; and preserve mobility over time, all while maintaining stability and safety. A device that is too stiff may exacerbate stress shielding and implant failure risks, whereas one that is too flexible may fail to adequately correct the deformity or maintain alignment over time. Implants capable of post-implantation adjustability would also allow clinicians to refine correction strategies based on individual growth patterns, alignment changes, or progression, potentially reducing the need for revision surgeries [32]. Several unmet and pressing clinical needs include the following:

• Adaptive correction: Current implants cannot respond dynamically to spinal growth or remodeling, which may lead to over- or under-correction over time, particularly in pediatric patients.
• Predictable long-term correction: Non-fusion systems such as VBT lack robust predictive models for long-term curve progression and mechanical failure, making outcome prediction and patient selection difficult.
• Minimizing revision risk: High rates of hardware failure or adjustment-related revisions in non-fusion techniques, and the irreversibility of fusion, underscore the need for implants that can be safely adjusted if necessary.
• Motion preservation without compromising stability: There is a clear gap for implants that combine effective spinal correction with preserved motion and minimal risk of adjacent segment degeneration or hardware fatigue.

**Figure 3:**
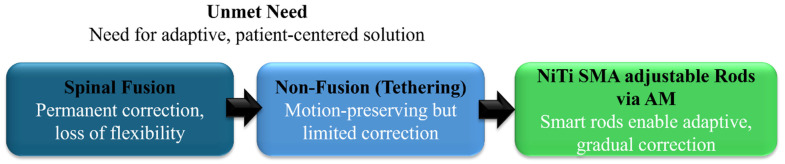
Evolution of scoliosis treatment toward patient-adjustable graded NiTi SMA rods.

These unmet needs motivate the development of a new paradigm in scoliosis treatment: one that integrates controlled, adjustable correction forces with dynamic motion preservation and patient-specific adaptability. For instance, researchers are exploring patient-specific spinal implants constructed from functionally graded Nitinol (NiTi) shape memory alloy rods (Figure 3) to fulfill these criteria [33,34,35]. By varying flexural stiffness and phase transformation behavior along the rod’s length, a functionally graded NiTi-SMA rod could provide rigid support in some spinal regions while remaining flexible in others [36,37]. Such an implant is envisioned to undergo post-implantation adjustments (e.g., via targeted heating or stress application), harnessing NiTi superelastic behavior and shape memory properties to exert continuous, tailored corrective forces while preserving segmental motion. In essence, this approach aims to unite the long-term stability of fusion with the adaptability of growth modulation, directly addressing the unmet clinical needs that current treatments fail to meet [38].

**Figure 4:**
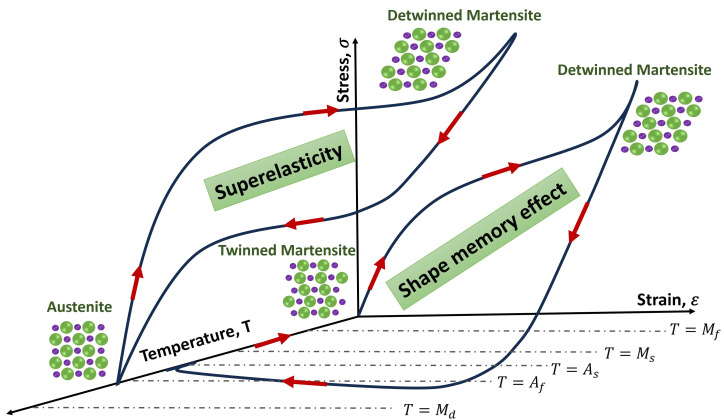
Illustration of the superelasticity and SME behavior of NiTi SMAs.

Shape memory alloys (SMAs), particularly Nitinol (an approximately equiatomic nickel–titanium alloy), have been explored in scoliosis correction since the 1970s [42]. These alloys exhibit a thermoelastic phase transformation that confers the shape memory effect (SME) (the ability to return to a pre-set shape when heated) and superelasticity (SE) (the capacity to undergo large reversible strains) [33]. Below the martensite finish temperature (M_f_), the alloy exists in a twinned martensitic structure, which can be readily deformed under modest stresses due to variant reorientation. Upon heating above the austenite finish temperature (A_f_), a reversible transformation occurs from martensite to austenite, enabling the material to recover its original configuration, predetermined during thermomechanical training (Figure 4). This phenomenon, known as the shape memory effect (SME), allows the alloy to generate substantial recovery stresses upon constrained transformation, an essential feature enabling corrective forces in orthopedic and spinal devices. Such properties allow SMA implants to apply gradual, controlled corrective forces to the spine, in contrast to the instantaneous correction of traditional rigid rods [43]. Early studies demonstrated the feasibility of harnessing Nitinol’s shape recovery to straighten scoliotic curves, making SMAs a promising avenue for less invasive or even fusionless scoliosis treatments.

Nitinol is the primary SMA used in spine implants, valued for its excellent biocompatibility and unique mechanical profile [44]. The biocompatibility and corrosion resistance of NiTi alloys have been verified through extensive in vitro and in vivo investigations, supporting their safe integration with biological tissues [34,45,46]. SMA transitions from a low-stiffness martensitic phase to a stiffer austenitic phase at a tunable transition temperature, enabling devices that activate at body heat. In the austenitic state at ~37 °C, Nitinol exhibits pseudoelastic behavior with 6–8% recoverable strain, far exceeding the ~1% elastic range of stainless steel. This combination of a lower Young’s modulus and high elastic strain capacity allows SMA implants to maintain a near-constant corrective force over a range of deformation, ideal for continuously remodeling a scoliotic spine without exceeding safe stress limits [43]. Furthermore, Nitinol’s shape memory effect means a device pre-formed to the desired spinal alignment can be cooled and temporarily deformed for insertion and then regain its original shape (and exert corrective pressure) as it warms in situ [36]. These thermal–mechanical properties underpin the use of SMAs in dynamic spinal implants for scoliosis correction.

Owing to this unique combination of thermomechanical functionality and biological compatibility, NiTi alloys have found increasing use in spinal correction surgery. Several studies have utilized SMA rods to either induce or correct scoliotic deformities in animal and human models. Activation of these rods has been achieved by elevating their temperature to the transformation range using methods such as warm water immersion, electric current Joule heating, or passive activation via body temperature. In these applications, the recovery strain of the SMA rod generates controlled bending moments on the spine, assisting in gradual deformity correction. However, earlier implementations often resulted in abrupt changes in the Cobb angle, either intraoperatively or shortly after implantation, leading to potential neurological complications and tissue overstress.

Several surgical techniques have leveraged these SMA properties for scoliosis treatment. One approach uses a Nitinol memory rod, contoured to the patient’s curvature at low temperature and then gradually recovering a straight shape when warmed, thereby gently mobilizing and de-rotating the spine. In practice, a cold, malleable Nitinol rod can be fixed with pedicle screws across the curved spine and then allowed to be activated by body heat or external warming to exert a sustained corrective moment on the deformity [47]. Another technique is vertebral body stapling with SMA staples, in which a shape memory staple is applied across the anterior vertebral growth plates on the convex side of the curve via a minimally invasive thoracoscopic approach [48]. The NiTi staple’s tins are straightened in an ice bath for insertion and then spontaneously curve inward to a locked C-shape at body temperature, anchoring into adjacent vertebrae. This staple produces asymmetric pressure on the growth plate, slowing growth on the convex side and gradually guiding the spine toward a straighter alignment while the patient continues to grow. Notably, these SMA-based procedures are fusionless, aiming to correct the deformity while preserving spinal motion and growth, an appealing strategy compared to early spinal fusion [49]. The thoracoscopic staple technique, for example, avoids a long posterior incision, and patients typically recover with maintained flexibility in the unstapled portions of the spine.

Emerging clinical evidence suggests that SMA implants can achieve significant deformity correction with favorable safety profiles. In a landmark animal study, a 6 mm Nitinol rod produced a Cobb angle reduction from 40° to 11° in an induced scoliosis model when heated, without causing tissue injury or neurologic deficit [43]. Subsequent biomechanical and clinical reports indicate that using a Nitinol rod intraoperatively is a feasible method to attain gradual three-dimensional curve correction in humans [42], although Nitinol rods remain uncommon in routine practice due to their high cost and lower stiffness compared to conventional titanium or steel rods [50]. By contrast, Nitinol staples have seen clinical use as a fusionless growth-modulation strategy for young patients. A multi-center series reported that vertebral body stapling had success rates comparable to bracing in preventing curve progression for moderate (<35°) idiopathic curves, with approximately 80% of treated curves remaining stable or improved at 2–3-year follow-up [49]. No implant-related complications (such as staple migration or breakage) were observed in these cohorts, underscoring the biocompatibility and safety of the NiTi devices in vivo [51]. Long-term outcomes of SMA-based treatments are still being studied; however, early results indicate that shape memory implants can correct spinal deformity while preserving flexibility and growth potential, representing a significant advance in scoliosis treatment technology [33]. Continued research and follow-up of SMA implants will further elucidate their efficacy and durability, but current data highlight their promise as innovative tools for less invasive scoliosis correction. In the context of this review, functionally graded NiTi-SMA rods fabricated via additive manufacturing (AM) are proposed as a candidate technology capable of filling this gap, offering gradual, adjustable correction while preserving spinal flexibility and reducing long-term mechanical complications.

This review synthesizes established clinical and materials evidence and, where appropriate, introduces author-proposed conceptual frameworks and illustrative modeling constructs to define development objectives rather than validated design protocols.

## 2. Material Differentiation

In scoliosis correction surgery, several biomaterials, such as ultrahigh-strength stainless steel (UHSS), cobalt–chromium (CoCr) alloys, and titanium (Ti) and nickel–titanium shape memory alloys (NiTi-SMAs), are commonly used as rod materials. Titanium is favored for its excellent corrosion resistance, biocompatibility, and magnetic resonance imaging (MRI) compatibility, which makes it highly suitable for both adult and juvenile spinal deformity surgeries [52,53]. Its biocompatibility derives from a stable titanium oxide passive layer that minimizes corrosion and metal ion release, conferring outstanding long-term implant performance [54,55]. Ti rods produce fewer MRI artifacts compared to CoCr and stainless-steel rods, which can improve postoperative imaging quality, although the clinical impact of this difference is still under investigation [56,57]. Notably, CoCr implants create slightly larger MRI artifact zones (on the order of a few millimeters) than Ti implants [57], but these artifacts generally do not impede visualization of neural structures on postoperative scans. Biomechanically, Ti rods have a lower elastic modulus (~110 GPa) than stainless steel (~200 GPa) and CoCr (220–230 GPa), which results in less stress shielding and better mimicry of natural bone behavior [58]. Despite its lower stiffness, Ti demonstrates excellent fatigue resistance and spring-back capabilities, meaning it can maintain shape after bending during surgery better than SS and CoCr [59,60]. However, Ti rods are more susceptible to fatigue failure under torsional loads compared to stainless steel [61]. In practice, titanium’s lower stiffness allows for more load-sharing with the spine (reducing bone stress shielding), but its relatively high yield strength (often 800–1000 MPa vs. 792 MPa for 316L stainless steel) means it can sustain large elastic deformations without permanent set [54,62]. This combination of flexibility and strength underlies the popularity of Ti-alloy rods, though care is needed with extreme contouring, as excessive twisting can still predispose Ti rods to fatigue crack initiation more so than stiffer steel rods.

CoCr alloys provide the highest stiffness and yield strength, which translate into greater corrective forces during spinal deformity correction, although this comes at the expense of a higher elastic modulus that may increase mechanical mismatch with bone and potentially exacerbate stress shielding effects [63]. For example, modern CoCr rods can exhibit yield strengths up to 2000 MPa, far exceeding those of Ti or SS rods [62]. This superior strength and rigidity allow CoCr rods to apply aggressive corrective torques and to use smaller diameters for the same strength, but they also concentrate stresses at the bone implant interface. Studies [64] comparing rod performance have shown that while CoCr and UHSS rods exert up to 42% higher corrective forces than Ti rods, Ti rods maintain their original shape better after implantation, with 90% shape retention compared to 54–77% for CoCr, SS, and UHSS rods. Moreover, intraoperative rod bending can introduce “notch effects” that reduce fatigue life, with CoCr rods demonstrating a 25% higher endurance limit than UHSS, SS, or Ti rods [60,65]. In clinical contexts, the increased rigidity of CoCr constructs has been associated with a higher incidence of proximal junctional kyphosis (adjacent segment failure) in adult deformity patients [66,67], presumably due to abrupt stiffness transitions that focus stress above the fusion. Some evidence also suggests that stiffer rods (CoCr or larger-diameter Ti) may achieve better thoracic kyphosis restoration in scoliosis surgery, supporting their use for severe deformities.

From a clinical perspective, studies indicate that CoCr rods may provide superior coronal plane correction and better kyphosis restoration compared to stainless steel rods [68,69]. However, postoperative rod deformation is observed even with stiff 5.5 mm CoCr rods, similar to the deformation seen in Ti rods, indicating that rod material alone cannot completely prevent shape changes after implantation [70,71]. Traditional SS rods, while high in stiffness and strength, tend to produce significant imaging artifacts and carry a greater risk of long-term corrosion or metallosis (e.g., elevated blood chromium levels), so they have largely been supplanted by Ti and CoCr in modern practice [72,73]. Nickel–titanium shape memory alloys (NiTi-SMAs) present a novel approach by combining a lower elastic modulus (30–70 GPa) closer to bone with unique shape memory and superelastic properties that enable dynamic, reversible deformation and gradual postoperative curve correction [74]. NiTi’s superelasticity allows it to undergo considerable strain and then recover its original shape when unloaded or heated, meaning that an SMA rod can continue to apply corrective forces over time as it attempts to resume a pre-set shape. NiTi rods act as dynamic implants that can adapt to patient motion and growth, unlike static steel or Ti rods. Mechanically, NiTi rods have shown comparable bending stiffness to Ti rods in cadaveric models but significantly higher torsional yield strength and energy absorption capacity [75]. This translates to a rod that can withstand larger twist deformations (up to 220% higher torsional yield in one study) before permanent damage. NiTi-SMA rods thus offer a promising future direction for patient-specific spinal fixation, potentially overcoming limitations of conventional rigid systems by enabling minimally invasive, adaptive scoliosis correction. Early biomechanical tests indicate that NiTi rods may exhibit superior fatigue performance; for instance, a memory-metal rod system endured 50% more loading cycles than a comparable Ti rod before failure. Clinically, a recent trial in adolescent idiopathic scoliosis reported that SMA rods achieved equivalent curve corrections to standard Ti rods over 5 years, with no device-related complications, demonstrating their safety and efficacy as a definitive implant [36,76,77]. The NiTi alloy’s high nickel content does introduce biocompatibility considerations (nickel ions can leach out if the protective oxide is damaged), but surface treatments (oxide coatings, etc.) can greatly enhance corrosion resistance and mitigate nickel release [78,79,80,81]. Ongoing research in surface engineering and functional grading of NiTi aims to optimize its biomechanical behavior while ensuring biological safety. NiTi-SMA rods combine bone-mimetic flexibility with the ability to deliver prolonged, gradual corrective forces, marking a compelling material innovation in spine surgery.

Table 1 compares key properties of Ti, CoCr, SS, and NiTi rod materials in spinal implants. Ti and CoCr represent two ends of the stiffness spectrum (lower vs. higher Young’s modulus), while NiTi offers a compliance closer to bone along with unique superelastic behavior. The values highlight how NiTi’s lower modulus and high elastic strain capacity contrast with the much stiffer behavior of CoCr and SS [62,82]. Notably, CoCr’s exceptional strength comes with a wide range of fatigue performance, whereas NiTi, despite its lower stiffness, can sustain high-cycle loads due to transformation-induced damping (with run-out beyond 5 million cycles in laboratory tests) [75]. These material differences have direct implications for construct rigidity, MRI imaging artifacts, corrosion profiles, and biomechanical load-sharing in scoliosis surgery, as discussed above [54,83].

## 3. Concept of Patient-Specific NiTi-SMA Rods

NiTi’s clinical value for patient-specific constructs rests on three pillars: (i) a tunable thermoelastic martensitic transformation that provides large recoverable strain and near-constant recovery stress over clinically relevant deformations; (ii) a lower effective stiffness (vs. stainless steel/CoCr) that improves load sharing and reduces stress shielding in the spine; and (iii) high-cycle durability with intrinsic hysteretic damping, which attenuates transient loads during daily activities [99]. In contemporary orthopedic devices, these same attributes are exploited for controlled deployment and sustained force delivery (e.g., staples, nails, and motion-preserving systems), establishing a mature translational pathway for NiTi components in load-bearing spine constructs.

### 3.1. Design Rationale and Technical Motivation

**Figure 5:**
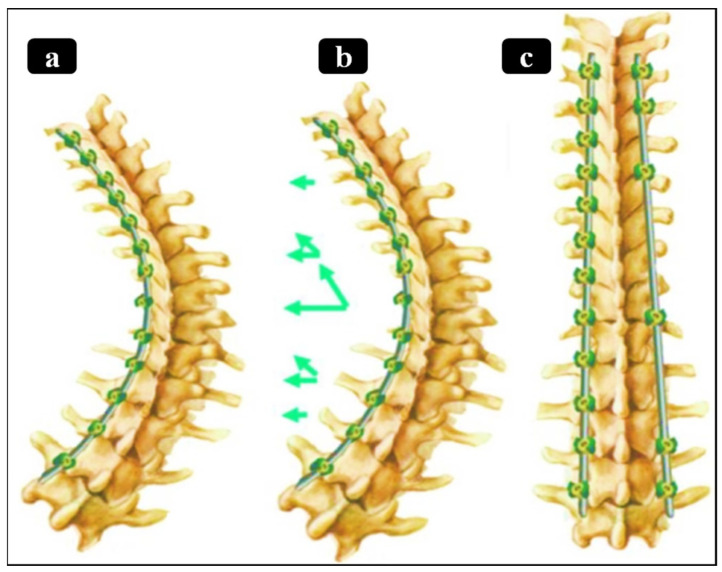
A Nitinol rod implanted in a spine: (**a**) deformed state to match a scoliotic spine, (**b**) heating the rod, (**c**) shape recovery and restoring the natural spinal curvature. During this recovery, the rod exerts axial torque and pulling forces on the concave side, achieving vertebral derotation and alignment correction [36,99]. (Green arrows shows the direction of axial torque and pulling forces).

The principal shortcoming of conventional scoliosis implants is their inability to adapt over time to spinal growth, remodeling, or physiological changes. Rigid spinal fusion hardware delivers immediate correction but permanently constrains segmental motion and cannot respond to ongoing vertebral growth or changes in spinal biomechanics. Conversely, tethering systems or growth modulation devices offer some mobility, but their correction depends heavily on unpredictable biological processes and may deteriorate due to mechanical fatigue or growth plate variability. Patient-specific NiTi-SMA rods offer a fundamentally different strategy: by harnessing the temperature or stress-induced martensite phase transformation inherent in NiTi alloys, these rods can deliver corrective forces in a controlled, gradual fashion while maintaining flexibility and accommodating physiological motion. The temperature-induced martensite phase transformation enables the rod to recover a predetermined shape when exposed to appropriate stimuli, exerting corrective forces aligned with the anatomical target. Stress-induced martensite phase transformation deformation ensures that the rod can undergo moderate to large in vivo loading without permanent deformation, thus preserving motion and damping transient biomechanical loads (see Figure 4a–c). This dual capability enables an SMA-based rod to serve as a semi-active correction system, capable of dynamic response rather than static fixation, potentially offering both corrective and protective functions. This concept is informed by prior work in which NiTi rods were used intraoperatively for scoliosis correction and subsequently replaced with rigid rods; however, few studies have exploited SMA rods for long-term, adjustable spinal correction [36,100,101,102]. These SMA devices can remain mostly passive during instrumentation. Subsequently, the active corrective process is triggered by external stimuli (thermal or mechanical) to a pre-programmed shape, allowing secondary correction over time (Figure 5). Because corrections can be made iteratively and away from vulnerable tissues, this concept might reduce the stress on soft tissues, improve postoperative comfort, and avoid abrupt neurological risk.

These properties can be tuned via composition and processing. Medical-grade NiTi is typically heat-treated to set its recovery shape and to establish transformation temperatures near body conditions. Small compositional shifts have large effects: even a ∼1 at.% increase in Ni depresses A_f_ by several degrees, enabling body-temperature activation. Likewise, alloying additions (e.g., Cu, Cr, and Fe) can adjust hysteresis width and plateau stress [103]. In practice, a scoliosis rod could be trained in terms of thermomechanical functionality to a pre-set curvature matching the desired spinal alignment. During implantation, the rod may be cooled or mechanically straightened to fit the current spine geometry; it then exerts corrective pressure as it warms up in vivo or is externally activated. Because NiTi’s Young’s modulus (30–50 GPa in austenite) is much closer to that of cortical bone (0.5–20 GPa) than stainless steel or CoCr alloys [104,105,106], the graded-stiffness effect also helps distribute loads more physiologically and minimizes stress shielding. In summary, NiTi’s combination of shape memory, superelastic plateau, and low stiffness provides a compelling material basis for a self-adjusting spinal rod. Importantly, NiTi’s fatigue and damping behaviors favor dynamic implants. When cycled through shape recovery or superelastic loading, NiTi dissipates energy via phase hysteresis, acting like an internal shock absorber. Instead of accumulating plastic strain under cyclic bending, NiTi will cycle along closed hysteresis loops with only small functional degradation (so-called functional fatigue). In bench tests and spine constructs, NiTi elements have demonstrated very high cycle lives (often millions of cycles) before failure [107,108]. One rough estimate is that NiTi can withstand ten- to one-hundred-fold more cycles than similar Ti rods under comparable loading, owing to this phase transformation damping. This high fatigue resistance suggests that a NiTi rod could adapt to growth and motion over many years without breaking while steadily delivering pre-programmed corrective forces. Moreover, NiTi’s protective oxide and capacity for surface treatments mitigate nickel ion release, ensuring biocompatibility even under cycling. Overall, these material attributes justify using NiTi for a long-term implant that is simultaneously flexible and self-actuating [109,110].

Material knobs that directly map to clinical goals include (a) transformation temperatures (A_s_/A_f_) set around 36–37 °C for autonomous actuation or staggered across segments for staged correction; (b) plateau stress and hysteresis width tuned by subtle composition/processing changes to deliver quasi-constant torque without overloading bone/anchors; (c) apparent modulus tailored by geometry (solid vs. porous/lattice) to better match local spinal compliance; and (d) surface state/finish qualified for corrosion resistance and ion-release performance. These choices mirror current device practices across NiTi orthopedic systems and align with expectations for body temperature characterization of Nitinol components. Clinically, NiTi’s superelasticity enables constructions that stabilize while preserving motion, reducing the need for very rigid fixation in select indications. Coiled-rod and loop NiTi elements (paired with Ti pedicle screws) have been deployed as dynamic stabilizers that provide physiological stability in flexion/extension and lateral bending, with encouraging early outcomes, an instructive analogue for designing patient-specific rods that supply sustained, sub-maximal corrective moments rather than abrupt, peak loads.

NiTi’s unique combination of lower stiffness and high recoverable strain enables near-constant corrective forces over large deformations. In the austenitic phase at 37 °C, Nitinol can undergo 6–8% recoverable strain (vs. 1% for stainless steel), allowing an NiTi rod to sustain bending loads without yielding. This property means that a NiTi rod can deliver a sustained bending moment across physiological motion, ideal for continuously remodeling a scoliotic curve. Crucially, NiTi’s one-way shape memory effect allows a rod pre-formed to the desired curvature to be cooled and deformed for insertion and then to recover toward its programmed shape as it warms in vivo, applying gradual corrective pressure. Clinically, this concept has been demonstrated: for example, a 6 mm NiTi rod was contoured to match a patient’s ideal spinal curvature, cooled in ice for malleability, and secured via pedicle screws on the concave side of the curve. As the rod warmed to body temperature, it slowly straightened toward its pre-set geometry, producing gradual derotation of the vertebrae; once optimal alignment was reached, the NiTi rod was exchanged for a rigid titanium rod. This approach yielded controlled corrective forces and lowered stresses on instrumentation, highlighting the potential of NiTi rods for semi-active scoliosis correction.

### 3.2. Mechanisms of Adjustability: Thermal Versus Mechanical Activation

There are two principal mechanisms by which an SMA rod might be adjusted post-implantation to achieve desired spinal correction:

1. Thermal Actuation: By selectively heating the NiTi rod (e.g., via electromagnetic induction, resistive heating, or other minimally invasive methods), the rod transitions from the martensite phase to austenite. This phase transformation triggers shape recovery that drives corrective forces. The temperature range for activation, the rate of heating, and the target recovery shape can all be tuned, thereby enabling staged adjustments over time. For example, a preliminary study on rabbit spines demonstrated noninvasive electromagnetic induction to gradually deform the spine model using SMA rods heated postoperatively to 34–47 °C [111].
2. Stress-Induced Superelastic Transformation: Alternatively, SMA rods operating above A_f_ can harness stress-induced martensitic transformation to generate corrective moments in response to mechanical loading [112]. This mechanism potentially allows the implant to respond dynamically to physiological motions, distribute loads adaptively, and mitigate stress peaks that contribute to hardware failure or adjacent segment degeneration.

The relative contributions of thermal vs. mechanical activation depend on the design of the rod (e.g., alloy transformation temperatures, cross-sectional geometry, and boundary conditions at attachment points), as well as the desired treatment timeline (immediate vs. gradual correction). Properly setting transformation temperatures (M_s_, M_f_, A_s_, and A_f_) and transformation stresses is critical to ensuring that adjustments occur at appropriate times and under safe thermal/mechanical conditions. Thermal activation must be carefully controlled to avoid local overheating or damage to surrounding tissues. Likewise, mechanical activation via superelastic cycling introduces fatigue loading and potential transformation temperature drift over time, both of which must be accounted for in design. Previous work using NiTi rods intraoperatively showed minimal acute neurologic disturbance, likely because the correction process was gradual rather than instantaneous [34,36].

NiTi rods can be repeatedly activated through controlled heating above A_s_/A_f_. In practice, various energy-delivery methods (induction coils, embedded resistive elements, and ultrasonic transducers) can heat the implant locally. For instance, one in vivo study used a 100 kHz induction coil to warm an implanted NiTi rod (A_f_: 34–47 °C) in a rabbit, achieving gradual spinal correction without thermal injury [111]. By segmenting the rod into zones with a slightly different A_f_ (e.g., by composition), one could selectively activate parts of the rod sequentially. For example, a lower A_f_ segment near a milder curve could straighten first under modest heating, while a higher A_f_ segment at the main curve apex would remain passive until given a stronger stimulus. This allows for tailored force application: the clinician could dial in curvature correction region by region and revisit older curves as needed, without re-opening the spine. Thermal cycling in NiTi produces internal damping with only modest metal fatigue; however, sustained temperatures must remain below approximately 45 °C to avoid neural and muscular injury [111,113]. Within these limits, thermal actuation provides a non-mechanical, physician-controlled means of adjustment. At physiological temperature, the rod may simultaneously operate in a superelastic regime. If the NiTi is fully austenitic at 37 °C (Af < 37 °C), routine spinal motion and corrective maneuvers can induce stress-driven martensitic transformation. Under bending or axial torsion, the rod will exhibit a pseudoelastic plateau: the moment remains nearly constant while the spine bends (stress-induced detwinning of martensite) and returns to the original shape when unloaded [106]. This behavior spreads loads over a range of displacement, in contrast to a linear spring. For typical medical NiTi, the transformation stress lies in the few-100 MPa range; once activated, the rod will accommodate large deformations (on the order of 5–8%) at this constant moment. The result is effective dynamic support; the rod self-adjusts as the patient moves or grows. Small physiological loads are absorbed elastically, while larger corrective bending drives the transformation to apply torque toward the memorized shape. This superelastic cycling provides internal shock absorption and energy dissipation via hysteresis [99,103]. Over many cycles, the rod will undergo minor functional fatigue (with a slight reduction in recovery moment), so designs must include safety factors. Therefore, stress-induced actuation makes the implant continuously responsive: it exerts corrective force whenever the spine attempts to deviate from the target alignment, without requiring any external trigger.

Thermal actuation (on-demand re-programming). Selective heating above A_f_ (e.g., short, localized energy delivery) triggers shape recovery and can be executed with safety envelopes that limit tissue temperatures to the low 40 °C for brief periods; in orthopedic NiTi staples, fixation is achieved by heating to 35 °C (shape memory route), and superelastic variants engage upon release, offering an instructive clinical analogue for establishing activation windows in rods designed for stress-induced superelastic actuation with continuous self-adjustment. When A_f_ ≤ 37 °C, the rod remains austenitic in vivo and responds to physiologic bending with a stress-plateau transformation (exerting an essentially constant moment over sizeable deflections and returning to its memory profile upon unloading), thereby smoothing load peaks at the bone implant interface (a behavior leveraged clinically in NiTi-based dynamic stabilization).

### 3.3. The Advantage of Functionally Graded (FG) Designs

A core innovation in the proposed rod concept is the use of functionally graded (FG) NiTi structures, whereby material properties (such as stiffness, transformation temperature, and geometry) vary spatially along the rod’s length or cross-section. FG design enables several technical advantages:

• Nonuniform force distribution: Regions requiring stronger bending or corrective force can be fabricated with higher stiffness or higher activation temperature (Af), while other regions remain more flexible. This spatial tuning allows the corrective moment to be focused where needed, reducing undue stiffness in regions where flexibility is desirable.
• Reduced stress concentrations and interface failures: Functionally graded stiffness reduces abrupt mechanical mismatches at screw–rod or tether interfaces, thereby lowering local stress concentrations that commonly lead to fretting, fatigue failure, or hardware loosening.
• Spatially optimized phase transformation: By grading transformation temperatures along the rod, phase transformation (martensite ↔ austenite) can be orchestrated to occur preferentially in segments according to anatomical curvature or corrective timing. This minimizes hysteresis and transformation mismatch, potentially improving fatigue life and limiting cycle stability.
• Tailored heat activation profiles: Segment-specific activation temperatures allow for localized thermal actuation; the clinical practitioner could selectively activate rod segments based on curvature severity or growth stage. This opens the possibility for region-by-region correction rather than whole-rod actuation.

Recent studies have examined FG SMAs in simplified beam or microstructures, demonstrating that grading transformation temperature or composition can influence bending stiffness, transformation hysteresis, and fatigue behavior [114,115,116]. However, these concepts have not yet been extended to full-scale spinal implants, where multiaxial loading, corrosion, and fatigue must all be addressed. Despite these promising properties, the true potential of FGMs lies in their successful translation from lab models to actual implants. In the broader biomedical literature, functionally graded implants have already shown clear benefits in reducing stress shielding and enhancing integration with host tissue [117,118]. For example, additively manufactured Ti-6Al-4V scaffolds with continuously varying porosity can tune the elastic modulus to match that of cortical bone (15–20 GPa) while preserving high strength [118]. In graded hip implant designs, tailoring the lattice density produced an optimized structure (with pore sizes 400 μm) exhibiting an elastic modulus of 15.7 GPa and compressive strength 530 MPa (values close to trabecular bone), thereby promising substantial reductions in stress shielding. Similarly, functionally graded Ti-hydroxyapatite (Ti-HA) dental implants have demonstrated markedly improved osteogenesis: specimens with graded HA content achieved significantly higher bone volume fractions (BV/TV) in vivo compared to homogeneous Ti implants [117]. These outcomes arise because graded composites transition stiffness gradually into the bone, steering load into the host tissue. In effect, smoothly varying material properties focus mechanical strain on the surrounding bone rather than on rigid hardware [118]. By analogy, a NiTi scoliosis rod with a corresponding stiffness or transformation gradient could distribute corrective forces more evenly along the curvature, matching the spine’s geometry and mitigating peak stresses at the pedicle rod interface. This bone-mimetic strategy, validated in other orthopedic contexts, suggests that FG NiTi rods would similarly improve force sharing, reduce implant loosening, and enhance integration.

Moreover, functional gradation can be built directly into the SMA’s phase-transformation behavior. NiTi’s one-way shape memory and superelastic effects can themselves be spatially tuned: compositionally or microstructurally graded NiTi alloys exhibit extended transformation ranges and multi-stage actuation that are impossible in uniform materials [119]. For instance, researchers have fabricated NiTi strips with a nickel content gradient or variable annealing profile and observed that different segments transform at different temperatures or stresses, producing a cascade of deformation [100,119]. One study demonstrated that local annealing of a NiTi rod creates a distinct soft–stiff interface: a 10 min Joule-heating pulse restored superelasticity in the heated section, yielding a rod with a compliant, low-stress gradient zone and a stiffer annealed zone [100]. Practically, such grading means that portions of the rod could activate under load in sequence. For example, a lower A_f_ segment might undergo phase transformation and provide corrective force early (e.g., during initial bending), while a higher A_f_ region remains passive until later (as forces increase with further adjustment or growth). This staged actuation mimics a built-in gradual rod: instead of a single, abrupt snap through, the rod supplies progressively increasing moments tailored to the patient’s curve severity. Importantly, graded NiTi actuators also exhibited novel multiway memory behavior under stress-free cycling, indicating additional recoverable deformation modes induced by thickness gradients [119]. The net effect is that FG SMA rods can modulate both where and when corrective forces develop, smoothing the overall force profile.

**Evidence Level and Scope Clarification:** To avoid ambiguity regarding translational readiness, the concepts discussed in this review are intentionally presented across different levels of evidence. Clinically established elements include current fusion and non-fusion scoliosis treatments, standard spinal fixation hardware, and their documented outcomes and complications. Theoretically plausible elements are those supported by established shape memory alloy thermomechanics, heat-transfer principles, and bench-scale or preclinical studies, including superelastic load sharing, transformation-temperature tuning, and functionally graded stiffness enabled by additive manufacturing. In contrast, concepts such as in vivo thermal activation strategies, spatially controlled actuation along the rod, postoperative modulation of corrective forces, and sensor-assisted control frameworks remain developmental and are presented as open research challenges rather than validated clinical solutions. Accordingly, the subsequent sections are intended to synthesize existing knowledge, highlight safety-critical gaps, and define development objectives, rather than to prescribe an immediately deployable clinical workflow.

## 4. Biomechanical Implications of Graded Topologies

Functionally graded structures (FGSs) are characterized by spatially varying composition, microstructure, or porosity and produce graded changes in mechanical or functional properties across a component. This gradation can help to reduce sharp material transitions, minimize stress concentration, and tailor mechanical or thermomechanical performance in a location-specific manner. In orthopedic biomaterials, FGSs are often used to bridge mechanical mismatches between implant and bone, reduce stress shielding, and improve implant-host integration [120,121,122]. When applied to spinal implants, graded structures allow for strategic variation in stiffness, transformation temperature, or corrective force delivery along the length of a rod, enabling spatial control of bending, force transmission, and flexibility. This approach is especially useful in scoliosis correction, where adjacent vertebral segments often present different curvature demands and biomechanical constraints.

In scoliosis implants, mismatches in stiffness between the rod and vertebrae, or abrupt transitions in rod rigidity, can lead to concentrated stresses at fixation interfaces or adjacent segments [66,123]. Functionally grading rod stiffness or geometry can smooth mechanical transitions and distribute corrective forces more evenly. Recent finite element studies on functionally graded pedicle screws, for instance, demonstrate reduced strain on vertebral bone and lower risk of screw loosening [124]. These findings suggest that grading can mitigate implant-induced stress shielding and mechanical failures. By designing graded stiffness zones, implants can be “soft” near flexible spine segments and stiff near apex regions needing stronger corrective torque. This spatial tuning promotes better force matching with vertebral compliance and may reduce compensatory loading on adjacent spinal segments.

Graded transformation temperature (A_f_/M_s_) profiles or stiffness gradients may also influence where and when a NiTi SMA rod enters martensitic or austenitic phases under physiological loads. If activation temperatures or transformation stress vary along the rod, phase transformation can be localized to specific zones. This localization could reduce hysteresis and transformation mismatch, potentially enhancing fatigue life by avoiding repeated cycling through full transformation in all segments. However, if not carefully designed, graded zones could also introduce mismatches in strain recovery, phase boundary movement, and thermomechanical cycling between adjacent segments, leading to complex internal stress distributions and fatigue hot spots. Some computational studies on graded porous or lattice structures in orthopedic implants show that zones with higher porosity or lower stiffness have reduced fatigue strength, and that graded transitions need to be optimized carefully to avoid early failure [125].

A graded SMA rod can be designed with varying transformation temperatures along its length to match the curvature profile of a patient’s scoliosis. For example, sections of the rod with lower A_f_ might activate earlier and provide initial corrective force, while higher-A_f_ sections remain passive until later stages (e.g., after growth or remodeling). This design enables staged correction: early correction of moderate curves, followed by more gradual correction of severe curvature as other rod segments activate. Grading the activation profile could serve as an internal program for time-dependent correction, reducing the risk of over-correction or neurologic insult by delaying or staging force application.

### Advanced Biomechanical Behavior and Interface Mechanics of Graded NiTi Rods

NiTi’s superelastic transformation inherently shapes its load-response. Under physiological bending and torsion, the alloy exhibits long, flat stress–strain plateaus that absorb energy and blunt stress peaks [126]. Graded thermal treatments can further tailor this effect: for example, localized Joule heating for 10 min fully restores superelasticity in the annealed rod section, creating a sharp mechanical gradient between heated and unheated zones [100]. Similarly, applying a thermal gradient anneal during processing produces a continuous variation in transformation temperatures along the rod, yielding a Lüders-like stress–strain profile with a built-in positive stress gradient [127]. These distributed phase-change behaviors allow a graded NiTi rod to engage its shape-recovery forces incrementally, smoothing the load curve and preventing abrupt stress concentrations on any single spine segment or screw. Functionally graded NiTi rods leverage these material effects to better match spinal biomechanics. By locally modulating phase-transformation behavior, a rod can be compliant in some regions (e.g., on the convex side of a curve) and stiff where needed (on the concave side), enabling controlled correction with preserved motion. In finite element models, a variable-stiffness NiTi rod combined with cable-screw anchors significantly reduced adjacent-disc pressures compared to rigid fixation, without raising stresses in the rod or screws. Each graded section of the rod begins to transform at different loads, effectively creating multiple yield stages. This multi-stage response distributes forces more evenly along the spine: one study showed that a stepped rod profile can carry load sequentially along its length, minimizing peak stresses in the fusion zone while allowing adjacent levels to move slightly.

In practice, NiTi’s adaptive compliance often yields healthier load transfer and motion preservation. In patients treated with NiTi implants without fusion, postoperative lumbar motion averaged 21° (versus 0° in rigid fusion) at 2.5 years [128], and no new adjacent-level degeneration was observed. This aligns with biomechanics: NiTi rods apply a steady corrective force that interacts with the spine’s viscoelastic growth properties, gradually realigning the curvature (consistent with Hueter–Volkmann growth modulation) rather than forcing an instantaneous correction [33,128]. As a result, corrective forces are disseminated to all the pedicle screws rather than concentrated on one [36]. In other words, the graded NiTi constructs share load across segments and enable micro-motion, leading to more uniform disc and facet loading. This shared, time-extended force profile may reduce complications such as screw loosening or junctional stress fractures that plague rigid constructs. NiTi’s intermediate stiffness and damping also favor a more physiological bone interface. With an elastic modulus much closer to cortical bone than stainless steel, NiTi rods transmit load to bone more effectively, reducing stress shielding and bone resorption [36]. In vivo studies support this: e.g., NiTi plates for fracture fixation produced slightly higher periscrew bone mineral density and volume than rigid cable-plate constructs (though differences were not always statistically significant) [129,129]. Importantly, no galvanic corrosion or adverse reaction was seen at the NiTi-titanium interface even after long-term implantation [129,129]. In contrast, highly rigid implants tend to offload bone and can cause local osteopenia. Moreover, compliant graded rods further mitigate adjacent-segment loading: one FE analysis of lumbar fusion showed NiTi and Ti rods raised adjacent-level disc stress by 12% versus intact, whereas an ultra-compliant polymeric rod avoided any stress increase [130]. By smoothing stress gradients at both the bone and disc levels, graded NiTi constructs can enhance osseointegration and reduce the progressive bone remodeling seen with rigid fixation.

Under cyclic spinal loads, NiTi’s phase-changing nature often improves durability. Fatigue cycling within the austenite–martensite plateau tends to stabilize the material and extend life [126]. Biomechanical tests confirm that NiTi constructs match titanium in static compression stiffness but have higher torsional yield (21.3 vs. 14.4 Nm) and toughness [75], meaning that they can absorb more multi-axis load before deforming. In one comparative study, NiTi and titanium rod constructs withstood similar fatigue cycles (181,000 vs. 64,000 cycles) before failure, indicating that both are viable for long-term use. For very high cycle counts, NiTi shows two distinct failure regimes: below 10^5^ cycles, cyclic martensitic transformations dominate crack initiation, whereas beyond 10^8^ cycles, failure becomes more stochastic [87]. Graded NiTi rods can be designed to operate primarily within the martensitic transformation range under expected loads, which, through martensite stabilization effects (and the energy-dissipating phase boundary), tends to push failure out to ultrahigh cycles [87,126]. The combined material and biomechanical advantages translate into favorable patient outcomes. Graded NiTi constructs provide adequate stiffness for spinal correction while preserving controlled motion, achieving mid-term corrective performance comparable to rigid fusion constructs. For example, patients stabilized with NiTi rods (without arthrodesis) reported significant improvements in Oswestry Disability and SF-36 and SRS-22 scores postoperatively, with outcomes explicitly noted to be better than those in a matched fused group [128]. Critically, these results were achieved with shorter surgeries and less blood loss, underscoring the clinical benefit of the self-adjusting correction process. By contrast, rigid fusion often sacrifices mobility and loads adjacent segments heavily. In summary, functionally graded NiTi rods redistribute loads more physiologically, spreading forces across screws [36], preserving segmental motion [128], and minimizing adjacent-disc pressure [127], which collectively reduces stress shielding and adjacent-segment stress. These features, supported by high-quality biomechanical and clinical data, highlight why graded NiTi devices hold promise for improved scoliosis treatment.

It should be noted that clinically established evidence exists only for current fusion and non-fusion scoliosis treatments, including their outcomes and complication profiles, and these data form the benchmark against which new concepts are evaluated. In contrast, SMA-related claims are primarily grounded in bench-scale and in vitro studies, supported by the well-established thermomechanical principles of NiTi (e.g., superelastic load sharing and transformation-controlled force delivery). At present, direct animal or clinical evidence demonstrating these effects in spinal applications is limited or absent, and extrapolation to in vivo scoliosis correction, therefore, remains theoretical. Accordingly, outcomes such as gradual correction or motion preservation are presented here as theoretically plausible, development-stage hypotheses, informed by material behavior and mechanical reasoning rather than validated clinical results. This distinction is intentional and critical to avoid overinterpretation of preclinical findings and to clearly delineate current knowledge from future research objectives.

## 5. Additive Manufacturing and SMA-Based FGS Implementation in Spinal Devices

The realization of graded NiTi rod designs depends critically on the ability to fabricate functionally graded components with precise control over composition, microstructure, and geometry. AM is a key to enabling technology in this context. AM techniques such as powder bed fusion–laser beam (PBF-LB) and directed energy deposition (DED) allow for variation in process parameters (e.g., laser power, scanning speed, and alloy feed composition) and geometry that can produce graded microstructures or thermal histories along a build. Recent reviews highlight that AM-based FGSs can reduce stress shielding, improve bone implant integration, and create implants that more closely mimic the gradient properties of bone [120,131,132,133]. In spinal implants, graded porosity or graded composition has been explored for interbody fusion cages, where porosity gradients improve load distribution and allow better osseointegration [121,125]. A few studies [134,135] propose graded metallic scaffolds or discs to mimic vertebral bodies or disc prostheses, suggesting that gradient transitions in stiffness or porosity can reduce implant subsidence and improve stress transfer. However, application of graded metallic SMA rods for scoliosis correction remains largely theoretical, and practical challenges, including downward grading of transformation characteristics, fatigue under cyclic bending, and manufacturing reproducibility, have not yet been fully addressed [33,34,136].

In this section, we provide a technical assessment of AM approaches for NiTi shape memory alloys and analyze how these processes influence the microstructure, phase transformation behavior, and functional performance of FG NiTi-SMA rods for scoliosis correction. We draw on the recent literature and process structure property insights to highlight key considerations, potential strategies, and unresolved challenges.

### 5.1. AM Techniques for NiTi: LPBF, DED, and Beyond

Several AM technologies have been applied to NiTi, each offering distinct advantages and limitations for SMA-based spinal implants. PBF-LB is the most widely studied technique for NiTi. PBF-LB offers high geometrical precision, customizable architectures, and the ability to fabricate fine-featured structures, which are attractive for patient-specific implants. However, the technique is sensitive to processing windows, particularly laser energy density, scanning strategy, and thermal gradients [137,138,139,140]. Directed energy deposition (DED) provides a more flexible approach for graded composition and geometries, allowing for tailored material feed and localized reheating. This can facilitate in situ property tuning but often leads to coarser microstructures, higher porosity, and increased thermal heterogeneity [141]. Hybrid or novel AM strategies, including in situ heat treatment, powder-feed modulation, or wire-based deposition, show promise for achieving location-dependent microstructural control and functional gradients [142]. The choice of AM method will strongly influence not only the geometrical fidelity of FG NiTi rods but also their phase transformation characteristics, mechanical behavior, and fatigue life, all critical factors for spinal implant performance.

**PBF-LB of NiTi SMA:** PBF-LB uses a focused laser to selectively melt NiTi powder layer by layer. This yields dense, net-shaped NiTi components with fine microstructures. Processing parameters (laser power, scan speed, and layer thickness) critically determine density and phase composition. Qu et al. [143] and Chernyshikhin et al. [144] showed that PBF-LB of 15–45 μm NiTi powder achieved > 99.5% density with moderate energy input (less than 160 J/mm^3^), producing a fully austenitic (B2) NiTi with transformation behavior close to bulk expectations. However, excessively high energy densities (>200 J/mm^3^) caused Ni evaporation and the formation of intermetallics, degrading superelasticity. In practice, PBF-LB can directly fabricate NiTi rods/spinal components with functional features; for instance, thin-wall and file-shaped NiTi parts have been printed for surgical tools [144]. PBF-LB is the most extensively studied method for AM NiTi for medical devices. It combines fine geometric integrity, high resolution, and process levers (e.g., hatch spacing, energy density, scan strategy, and build orientation) that enable texture control and (even more importantly for this work) the possibility of spatially tailoring properties across a component. These capabilities position PBF-LB as a leading platform for patient-specific, functionally graded NiTi rods that couple transformation-temperature (TT) gradients with engineered stiffness profiles. This perspective is consistent with the objectives outlined in the AM section of this review. Representative reviews and studies detail both the breadth of PBF-LB NiTi research and the state of the art in achieving defect-lean, superelastic components suitable for biomedical use [139,143,145,146]. A central technical risk for implant-relevant PBF-LB NiTi is process-induced chemistry drift: preferential Ni evaporation at high melt-pool temperatures and oxygen/carbon pickup from the powder/build atmosphere. Both effects disturb TTs, narrow or shift the superelastic window, and promote secondary phases (e.g., TiO_2_ and TiC) that deplete the matrix and compromise corrosion/fatigue resistance. Because NiTi’s TTs are acutely composition-sensitive (on the order of 10 °C per 0.1 at.% Ni), small Ni losses translate into clinically meaningful functional changes [147,148,149,150].

**Figure 6:**
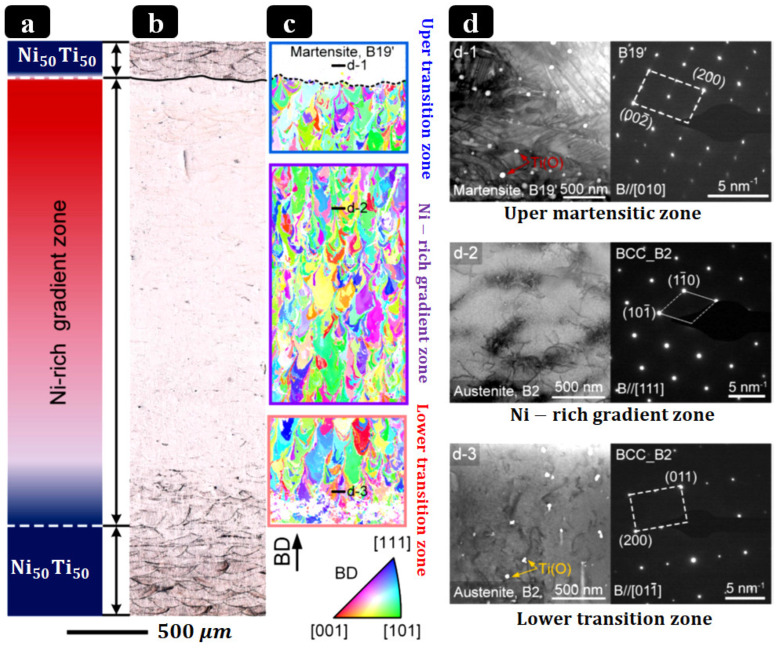
Compositionally graded NiTi produced by PBF-LB via in situ Ni addition: (**a**) schematic of Ni-rich → Ni-lean gradient; (**b**) optical microstructure; (**c**) IPF maps (build direction vertical) illustrating crystallographic variation along the gradient; (**d**) STEM images evidencing phase constitution in different zones [142].

Mitigation strategies encompass (i) parameter windows that avoid excessive energy density; (ii) slightly Ni-rich feedstock to offset Ni volatilization; (iii) strict oxygen control and powder-handling protocols; (iv) post-processing (stress relief/HIP/aging) to homogenize composition and stabilize transformations; and (v) surface finishing/coatings to suppress Ni release and enhance corrosion resistance. As process controls have matured, multiple groups have demonstrated room-temperature superelasticity in as-built or lightly treated PBF-LB NiTi, while surface engineering (e.g., solution + finish, anodic/oxide growth, and other coatings) significantly improves corrosion performance in simulated body fluids [146,151,152]. Beyond chemistry control, PBF-LB also affords crystallographic texture engineering: careful tuning of hatch/energy and thermal gradients can drive robust ⟨001⟩ fiber textures along the build axis, useful for directional stiffness/strain recovery in rod-like implants [153,154]. Functionally graded NiTi directly supports the design goals of patient-specific scoliosis rods: graded TT (A_s_/A_f_) and graded rigidity can concentrate corrective moments where needed while preserving flexibility elsewhere, reduce stress concentrations at screw–rod interfaces, and enable staged actuation during growth or remodeling. Recent experimental advances now provide concrete demonstrations: Zhu et al. [142] fabricated compositionally graded NiTi by adding elemental Ni during PBF-LB in situ, achieving a continuous 2.75 mm gradient (49.6–52.4 at.% Ni) with location-specific superelasticity/SME and TT profiles, a powerful proof of concept for AM-enabled grading relevant to spinal rods. These AM results sit within (and extend) the broader FG-NiTi literature, which has historically relied on diffusion annealing or laser surface annealing to create TT/stiffness gradients [155]. Figure 6 illustrates a PBF-LB method to compositionally graded NiTi via in situ Ni addition, yielding spatially tunable transformation temperatures and local superelastic response.

**DED of NiTi SMA:** DED processes feed wire or powder into a melt pool to build parts. This technique produces larger, near-fully dense NiTi components with a reletively coarse microstructure. For example, Silva et al. [156] demonstrated the µ-wire arc DED of NiTi using 0.3 mm NiTi wire, depositing sequential beads to create a wall. The DED method enables thick deposits (many mm) and multi-layer structures with robust interlayer bonding. DED can be extended with in situ alloying: by co-feeding pure Ni powder or wire along with equiatomic NiTi feed, one can create a continuous compositional gradient during printing. Zhu et al. [142] used PBF-LB with mixed NiTi and Ni powders to produce a 2.75 mm gradient zone where Ni content varied from 49.6 to 52.4 at.%. High cooling rates locked excess Ni into the solution, yielding location-specific functionality (see below). Similarly, hybrid strategies such as combining PBF-LB printing of a NiTi skeleton with subsequent heat treatment or cold spray can tailor the stiffness profile. Although still exploratory for NiTi, hybrid AM techniques (e.g., sequential deposition and machining, or multi-step thermal treatments) have been applied to metallic FGMs and could be adapted to NiTi for spinal implants [157]. Across all manufacturing routes for NiTi components intended for biomedical applications, the primary challenge lies in controlling carbon and oxygen contamination [158,159], as well as nickel evaporation, which can significantly alter the alloy’s mechanical and functional properties [160]. Depending on the fabrication method, the extent of these effects and the available control strategies differ. In fusion-based additive manufacturing processes, such as PBF-LB and DED, maintaining a strictly inert gas environment and carefully tuning process parameters can minimize oxygen and carbon uptake [96,161]. However, nickel evaporation during melting and solidification remains a persistent issue, potentially leading to deviations from the nominal composition and deterioration in shape memory behavior. Conversely, in sinter-based techniques, such as Binder Jet Additive Manufacturing (BJAM) [162,163], nickel loss is generally negligible, but carbon and oxygen contamination becomes more pronounced due to the presence of organic binders used during shaping and de-binding stages. Therefore, while additive manufacturing enables the design and fabrication of both structurally and compositionally graded NiTi systems, each technique requires specific optimization and customization to mitigate contamination and compositional changes according to the desired biomedical performance.

### 5.2. Process–Structure–Property Relationships in AM-NiTi

**Figure 7:**
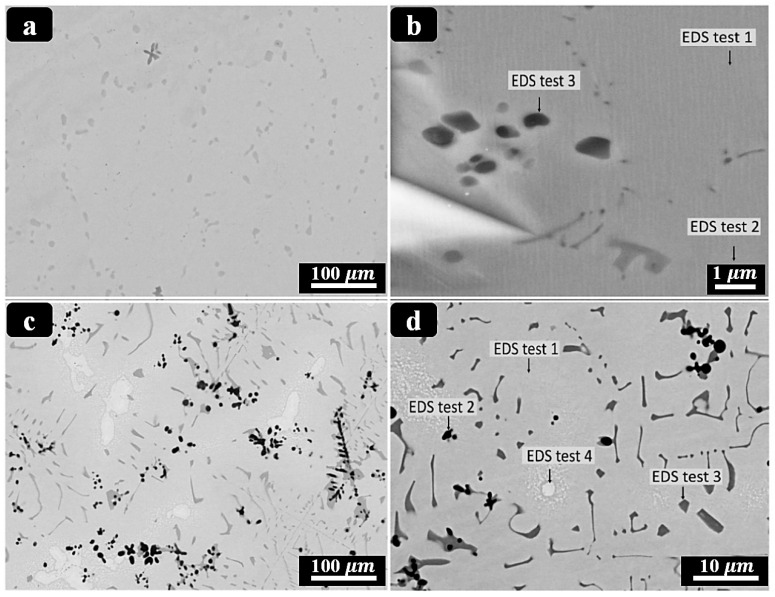
FESEM-BSE micrographs of the AM-fabricated NiTi rod cross-section: (**a**,**b**) first (lower) layer at low/high magnification, predominantly NiTi matrix with fine grains and small pores; (**c**,**d**) second (upper) layer, coarser grains and dispersed intermetallic phases Higher hardness (H) and modulus (E) were measured in the Ni-rich second layer than in the first [156].

The performance of AMed NiTi is governed by a complex interplay of powder feedstock, process parameters, and post-processing treatments, which together define the microstructure, phase constituents, transformation behavior, and functional behavior. These process–structure–property linkages are especially critical for FG NiTi rods, where local variations in composition, microstructure, and phase behavior must be precisely controlled to achieve graded transformation behavior and consistent actuation over the implant lifetime. Graded AM processing yields tangible material gradients in NiTi rods. For example, in the DED NiTi wall by Silva et al. [156], the first (lower) and second (upper) layers differed in chemistry and microstructure. The Ni-rich top layer contained more intermetallics (Ni_3_Ti, NiTi_2_, and Ti) and higher O/N content than the B2-NiTi matrix of the bottom layer. This produced a sharp jump in local mechanical properties at the layer interface. Instrumented indentation confirmed that the upper Ni-rich zone was significantly stiffer and harder: an average elastic modulus of 100 GPa and a hardness of 7 GPa, versus 75 GPa and 4 GPa for the lower NiTi-rich zone. Thus, the rod inherently possessed a graded modulus, the top zone bearing a higher load than the bottom. Figure 7 presents FE-SEM images of the cross-section from that study (Silva et al. [156]), illustrating the different microstructures. Figure 7a,b show the first layer (fine-grained, mostly B2-NiTi), and Figure 7c,d show the second layer (coarser grains, multiple phases). This vividly demonstrates how AM processing conditions created a functionally graded SMA microstructure.

These microstructural (Figure 7) gradients directly influence thermal–mechanical behavior. The Ni-rich regions have higher transformation temperatures and larger hysteresis than the NiTi regions. Only the second layer exhibited B19′ ↔ B2 martensitic transformations (with A_f_ above room temperature), whereas the first layer remained fully austenitic at all measured temperatures. The extra Ni and impurities in layer 2 raised its A_f_ by several tens of °C. In practice, this means an FG NiTi rod could have zones that are superelastic at 37 °C, adjacent to zones with higher Af. By adjusting local chemistry via AM, designers can tune the transformation window along the rod: for instance, ensuring mid-rod segments remain superelastic at body temperature while extremities (near fixation points) have higher stiffness or shape memory retention. Thus, the AM-induced compositional gradient delivers a graded transformation profile and stiffness profile. This graded functionality could be exploited to match each vertebral level’s mechanical needs. Table 2 summarizes AM-specific risks for FG NiTi rods, their impact on transformation behavior and fatigue, and practical mitigation/QA strategies.

**Clinical and Biomechanical Implications:** Functionally graded NiTi rods promise new biomechanics in scoliosis surgery. By spatially varying stiffness, an FGM rod can more evenly distribute corrective forces. Biomechanical simulations by Brailovski et al. [100] and others have shown that a monolithic rod with variable flexural rigidity reduces stress concentrations at rod ends and adjacent segments. For example, Joule-annealing a portion of a NiTi rod to restore superelasticity (softening that segment) created a smooth stiffness gradient along its length, which in FE models decreased adjacent-level motion and stress relative to uniform rods. Functionally, gradation helps avoid an abrupt stiffness mismatch (and the shear stresses it causes) that can lead to junctional problems. Additionally, NiTi’s superelasticity itself offers unique correction mechanics. Conventional spinal rods are bent into the scoliotic shape, applying elastic force on the spine. A pre-tensioned NiTi FGM rod could apply a distributed, progressive force. Yeung et al. [169] demonstrated that a superelastic NiTi rod (pre-contoured at low temperature and then warmed) generated a nearly constant recovery force within a strain plateau (8% strain range). In a goat scoliosis model, this resulted in gradual curve reduction over days postoperatively, as the rod continuously pulled the spine straighter. In practice, NiTi rods can thus be contoured at room temperature and then locked to exert gentle forces at body temperature. An FGM NiTi rod could be designed so that its central superelastic region delivers this controlled corrective force, while stiffer ends hold the construct rigidly. Clinically, this may translate into safer, more automated scoliosis correction with reduced intraoperative forces and improved preservation of spinal motion. The superelastic zone further provides a shock-absorbing, flexible function. A midsection of lower modulus can permit small movements without plastic deformation, potentially reducing stress at the bone–screw interface and improving patient comfort. Conversely, the higher-stiffness ends ensure firm fixation. Moreover, grading can mitigate stress shielding: if the rod gradually stiffens toward the ends, adjacent vertebrae may experience more natural load-sharing than with an abrupt Ti or steel rod. These biomechanical advantages (reduced junctional loads, continuous correction forces, and staged stiffness) all stem from the tailored phase behavior and elasticity of FG NiTi. Importantly, any Ni release concerns can be addressed with modern surface treatments (e.g., oxide or DLC coatings), as noted by Yeung et al. [169], and the biocompatibility of NiTi is well established under ASTM F2063 guidelines [170].

Freedom of AM invites the computational and experimental design of FG rods. Topology optimization and gradient design can specify spatial distributions of Ni content, porosity, or geometry to meet biomechanical criteria. For example, designers might use FEA to tailor a rod’s cross-section or internal lattice along its length so that stiffness or bending moment under load matches a desired profile. Combined with NiTi’s temperature-dependent superelastic response, this multi-parameter design space is vast. Early studies have already used parametric strategies: one study found that grading Ni content (via in situ AM) broadened stress–strain curves and lowered hysteresis [142], suggesting the design of self-adjusting rods. Future work could use generative algorithms (informed by FDA-recommended constant-life fatigue curves [109]) to optimize the rod for both static correction and long-term cyclic loading. Fatigue behavior is a critical consideration for spinal implants. NiTi is known to tolerate millions of superelastic cycles, but AM processing effects (porosity, surface finish, and phase distribution) require careful control. For instance, scaffolds of PBF-LB NiTi have shown promising endurance if surface defects are minimized, but even small unmelted particles drastically reduce fatigue life [171]. In graded rods, stress concentrators at internal transitions could become fatigue initiation sites. The FDA specifically notes that NiTi fatigue life depends sensitively on composition and processing [172] and recommends device-specific strain-life testing. Practically, this means a graded NiTi rod would need localized fatigue characterization (e.g., zones of the highest Ni content or stress concentration) and potentially surface polishing or post-build HIP (hot isostatic pressing) to eliminate defects. Reports suggest that NiTi printed by PBF-LB, when processed correctly, can meet medical fatigue standards [171]; however, caution is warranted since oxygen uptake during AM (from residual powders or atmosphere) can embrittle NiTi and shorten fatigue life.

Despite these challenges, the benefits in surgical utility are significant. NiTi FGM rods can be pre-bent or shape-set into ideal corrective shapes at room temperature, enabling minimally invasive insertion. At body temperature, their return force gradually realigns the spine, potentially reducing the need for forceful adjustment by the surgeon. Also, the graded design could allow a single implant to replace multiple rod segments: for instance, a composite rod with a soft middle and stiff ends may eliminate the need for additional springs or dynamic connectors. Intraoperatively, the ability to dial-in rod behavior (via heat treatment or AM pre-sets) could simplify customization for each patient’s curve severity.

**Regulatory and Practical Considerations:** Moving from prototype to clinic requires compliance with regulatory standards and reproducibility. NiTi medical devices are governed by FDA guidance and ASTM/ISO standards. Notably, ASTM F2063 specifies allowable NiTi chemistries, transformation temperatures, and mechanical properties [170,173]. While AM NiTi is covered under these, regulators will expect proof that the entire build (with gradients) meets biocompatibility (such as ISO 10993 [174]) and corrosion criteria. The FDA’s 2021 Nitinol guidance emphasizes several points relevant here: fatigue testing should use liquid environments (PBS), not air, employ strain-life (constant-life) fatigue curves rather than traditional stress-life plots, and be specific to the final device’s processing (i.e., test a sample built with the same AM parameters). These recommendations must be followed for any AM-made NiTi rod. In practice, a graded NiTi rod would need to demonstrate (for example) that its weld-bonded layers and compositional transitions do not induce premature fatigue or corrosion. Any post-processing (e.g., annealing or shape-setting) must be validated. Reproducibility of the AM process is also critical. PBF-LB and DED can have variability in powder feedstock and energy input that alter phase balance. For example, as noted, PBF-LB at very high energy leads to Ni volatilization [143]; similarly, in DED, the wire feed and arc stability affect oxygen pickup. Manufacturers would need tight process controls (and in-line monitoring) to ensure that each NiTi rod has the same graded profile. Standards like ASTM F3055 [175] (for AM metals) and the FDA’s recognized ASTM F2063 [170] would guide material testing and reporting. Mechanical integration challenges exist. A NiTi FGM rod must interface with standard pedicle screws (typically Ti or stainless steel). Dissimilar metal galvanic corrosion must be addressed (usually with insulating PEEK washers or coatings). The structural gradient also means that screw clamping forces might need adjustment; too much compression in a stiff zone could cause plastic deformation of the NiTi. Installation protocols would have to specify temperatures (for malleable shaping) and torques carefully. Fatigue limits, too, must be set; e.g., a NiTi FGM rod might permit a higher cyclic bending amplitude in its soft midsection, but that necessitates showing a safe fatigue factor under those conditions.

## 6. Key Considerations for FG NiTi-SMA Rod Development

**Figure 8:**
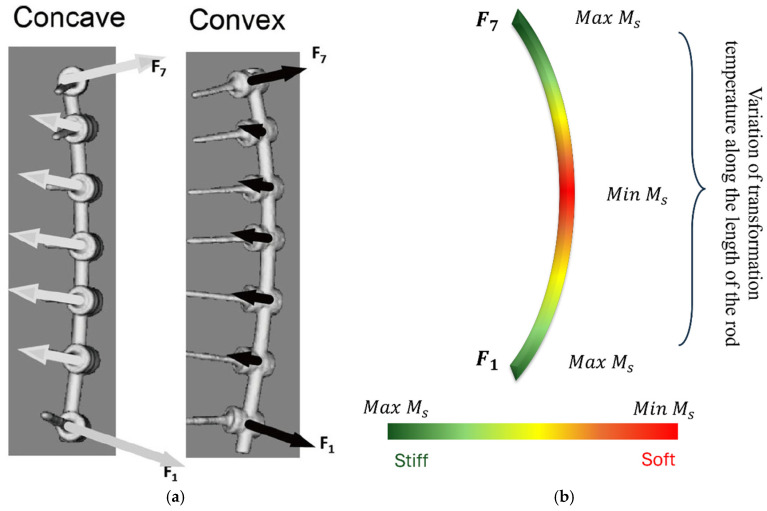
(**a**) Three-dimensional CT image of rod geometry before insertion and one week after surgery [178]; (**b**) conceptual design of functionally graded SMA rod to adapt the curvature of the spine in scoliosis case.

The successful development of patient-specific FG NiTi-SMA rods depends on delicately balancing biomechanical, thermomechanical, durability, and patient-centered adjustability considerations. Design of FG NiTi-SMA scoliosis rods must balance precise anatomic correction with adequate mechanical support. The rod’s memory shape should be pre-configured to the desired spinal alignment (normal coronal and sagittal curves) so that, on activation, it exerts controlled corrective forces [76,127]. To enable implantation, rods are typically cooled (e.g., in ice) to the pliable martensitic phase and contoured to the patient’s deformity [76]; on warming to near body temperature, the NiTi gradually reverts toward its austenitic memory shape. In practical terms, NiTi’s relatively low elastic modulus (typically 40–75 GPa vs. 110 GPa for titanium rods) allows for lower instantaneous implantation forces and reduced stress on bone–screw interfaces [76]. FG rods should, therefore, be designed with segment-specific stiffness profiles (e.g., softer near the curve apex; stiffer toward anchor ends), so as to maintain overall construct stability while preserving some residual motion [127]. In numerical models of an instrumented spine segment, a NiTi rod with deliberately graded flexural rigidity significantly reduced adjacent-disc pressures without increasing vertebral implant stresses, compared with a uniform rigid rod. Such functionally graded stiffness is expected to mitigate adjacent-segment degeneration (a known complication of rigid fusion) by allowing for more physiological load transfer (see Figure 8 for how TT tuning shifts mechanical responses) [176].

The rod diameter and cross-section must also be chosen to provide sufficient shape-recovery force without exceeding safety limits. In practice, prior NiTi scoliosis devices have used 5–6 mm diameter rods for pediatric/preadolescent patients. The NiTi alloy’s yield and plateau stresses (often 500–600 MPa for superelastic grades) determine the maximum corrective moment per bend. Design guidelines, therefore, require verifying that the programmed shape change yields the desired Cobb-angle correction within these stress bounds. At the same time, any pre-straining of the rod during contouring should remain within the elastic (martensitic) limit to avoid permanent damage. Overall, a careful kinematic analysis is needed to ensure that the NiTi recovery force will realign the spine as intended without causing screw pull-out or vertebral fracture. Notably, studies have shown that NiTi rods (used as temporary implants) can achieve large three-dimensional corrections by harnessing slow postoperative shape recovery: for example, NiTi wires produced an additional 40° Cobb angle correction over two weeks in a rat model [33]. In human applications, the adjustment timeline must be managed so that incremental correction (whether from gradual warming or other actuation) is paced safely as tissues relax and remodel. The material-level challenges of NiTi also demand attention. The unique thermoelastic transformation of NiTi (B2 ↔ B19′) is sensitive to composition, heat treatment, and defects. The transformation temperatures must be tuned so that the rod is fully martensitic (soft) at implantation (<20–25 °C) but mostly austenitic (stiff) at body temperature (37–40 °C). This requires tight control of nickel content (e.g., 50.5–51.5 at.% Ni for A_f_ 37 °C) and heat treatment to ensure a stable, single-phase austenite at physiological temperature. In practice, even small Ni losses (e.g., from evaporation) can raise A_f_ significantly [168,177]. For instance, PBF-LB processing with slow scan speeds was found to elevate A_f_ by 30 K due to preferential Ni evaporation. Additionally, NiTi may exhibit R-phase or multiple transformation peaks if not fully homogenized, which could complicate its actuation profile. Aging treatments (creating Ni_4_Ti_3_ precipitates) are often used to adjust transformation temperatures and stabilize performance, but these must be uniform through the rod to avoid local T discrepancies or training effects.

The rod’s stress–strain response under physiological bending, torsion, and axial loads determines its ability to apply corrective moments and adapt to spinal motion. In the superelastic regime (above A_f_), NiTi enables large, recoverable strains, but the transformation plateau stress and hysteresis shape influence how much corrective force is generated and how the implant reacts under cyclic deformation (see Figure 8). The graded design must ensure that each segment of the rod can undergo sufficient transformation without plastic deformation and that the corrective forces generated align with safe stress thresholds in vertebrae and fixation interfaces [178]. Achieving and maintaining spinal correction implies not only initial force application but also sustained corrective stability over time. This requires that the NiTi rod can keep delivering shaping forces without significant drift in phase transformation behavior or loss of corrective moment due to functional fatigue, creep, or plastic deformation. Additionally, graded stiffness designs must avoid unintended spring-back or force shifts as the spine remodels or as the patient grows, which could undermine long-term alignment. From a thermomechanical performance perspective, for spinal rod applications, it is critical that A_f_ is close to, but ideally slightly below, body temperature, allowing for body-heat-driven shape recovery without requiring excessive external heating. If A_f_ is too low, the rod may lose rigidity and corrective force prematurely; if A_f_ is too high, external heating may be required, making noninvasive actuation more difficult or dangerous. Small deviations in the Ni/Ti ratio or local microstructure (e.g., due to processing or aging) can shift these temperatures by tens of degrees [179]. In an FG design, spatial variation in A_f_ along the rod can enable staged or region-specific correction but also demands rigorous control of local transformation behavior. Each activation cycle (thermal or stress-induced) involves a phase transformation that can generate heat, strain, and phase boundary movement. Repeated cycling may lead to functional fatigue, characterized by shifts in transformation temperatures, reduced recoverable strain, or degradation of shape memory behavior. Studies have shown that NiTi fatigue behavior is temperature-dependent, and mechanical loading at elevated temperatures can accelerate degradation [180,181]. Moreover, graded rod sections may experience different thermal loads or cycling histories, which could lead to non-uniform functional drift across the implant.

Native NiTi has generally favorable biocompatibility due to its titanium-rich oxide surface layer [176]. In vitro, properly prepared NiTi (polished/electropolished) often shows corrosion resistance comparable to medical steel [182]. To guarantee durability and safety, the impact of corrosion behavior is important. NiTi implants are exposed to physiological fluids, mechanical wear, and cyclic transformations, all of which may impact corrosion behavior and ion release. Clinical studies [110,176,182] of NiTi rods retrieved from patients up to 1–2 years postop found no gross corrosion and no elevated Ni in blood. Nickel ion release is a particular concern, given nickel’s potential to elicit adverse biological reactions. Surface degradation during cycling, phase boundary formation, and microstructural changes may exacerbate corrosion. Studies indicate that aging treatments can influence corrosion resistance, with optimal aging conditions improving performance [183,184,185]. Therefore, developing corrosion-resistant surfaces or passivation strategies is essential, particularly for graded implants, where different segments may undergo distinct microstructural changes. For enhanced osseointegration and safety, additional coatings are often applied. A ceramic or polymeric overlayer can act as a Ni-barrier. For example, electrochemically grown TiO_2_ nanotube arrays (≈1 µm thick) have been demonstrated on NiTi; when filled or capped with biocompatible polymers, they block Ni diffusion and also resist bacterial adhesion [182]. Similarly, apatite or calcium–phosphate coatings (via plasma spray or pulsed laser deposition) can create a bioactive interface. One report described a nanoporous composite layer containing hydroxyapatite on an AM-fabricated NiTi surface (via pulsed-laser sintering), which markedly improved osteoblast attachment and early collagen formation [186]. Mechanical surface treatments also help: ultrasonic nanocrystal surface modification (a burnishing process) was shown to markedly reduce surface roughness and subsurface porosity on AM NiTi, yielding higher hardness and corrosion resistance [187]. Therefore, any strategy that thickens or stabilizes the outer TiO_2_ film (such as anodization or oxygen plasma) will reduce Ni ion flux. At the same time, creating a rough or porous outer texture (including intentional porosity from AM) can promote bone in-growth and mechanical interlock.

Finally, practical considerations shape clinical use. NiTi is essentially non-ferromagnetic (though slightly paramagnetic), so it is generally MRI-conditional at clinical field strengths [188]. In practice, Nitinol implants produce minimal heating and only minor artifacts on MRI or CT, like titanium. This contrasts with stainless steel, which creates significant image distortion. Thus, postoperative MRI of the spine is typically feasible with NiTi rods, a critical factor for patient follow-up. X-rays and CT imaging are likewise unhindered aside from the usual metal beam-hardening artifacts (no special shielding is required). Implantation of a NiTi FG rod requires a new workflow. The rod must be kept below its transformation onset temperature (often near 0–10 °C) during shaping. In practice, this means using ice or cold saline while the rod is locked into the screw heads and then applying a heat source (warm saline; radiant heat) after securing to gradually activate the shape change [76]. Operating room protocols must accommodate this (e.g., having cold sterile containers and warm-water irrigation ready). Surgeons also need to avoid overwriting the rod’s designed memory: once the desired correction is achieved, the actuator (temperature) must be controlled so as not to induce over-correction. Backup plans (such as placing a locking cap on the rod) are prudent if incomplete adjustment is reached. In terms of mechanical margins, the rod should be dimensioned with safety factors: for example, pilot testing may determine the maximum curvature and torque the rod can sustain without fracturing, which sets the clinical corridor of safe manipulation. Unlike one-time fusion implants, FG SMA rods may continue to exert forces after surgery. Clinical teams must plan the timing of any external triggers (e.g., heat application sessions) to maximize correction without harming tissue. Early rehabilitation (gentle movement) might assist gradual realignment, as was observed in animal experiments where daily activity co-operated with the rod’s memory effect [33]. If the concept includes patient-controlled adjustments (e.g., through wearable heaters or magnetically tuned devices), protocols must ensure the adjustment occurs only at body-safe rates (keeping local temperature below 45–50 °C to avoid thermal injury). In sum, the clinical integration of FG NiTi rods will require new guidelines for imaging, intraoperative techniques, and postoperative management to fully exploit their adaptive behavior while maintaining safety.

**Critical Perspective and Evidence Scope:** Taken together, the advantages, limitations, and challenges discussed across material differentiation, biomechanical implications, and additive manufacturing considerations must be interpreted within a clear evidence hierarchy. Clinically established outcomes currently exist only for conventional fusion and non-fusion systems, which, therefore, serve as the appropriate benchmark. In contrast, FG NiTi-SMA rods represent a preclinical, development-stage concept, supported by well-established SMA thermomechanics, emerging AM capabilities, and bench-scale or modeling studies, but not yet by animal or clinical validation. Relative to soft shape-morphing and SMP-based systems, FG NiTi rods occupy a distinct niche as load-bearing implants capable of sustained corrective force delivery and superelastic load sharing while also introducing unique challenges related to manufacturing control, fatigue, thermal safety, and regulatory complexity that are discussed in detail earlier. Accordingly, the FG NiTi approach should be viewed neither as a direct replacement for existing technologies nor as universally superior but rather as a complementary and adaptive design space whose clinical value depends on the successful integration of materials design, fabrication control, and conservative actuation strategies.

## 7. Conceptual Design Framework and Development Roadmap for Patient-Specific FG NiTi-SMA Rods

**Figure 9:**
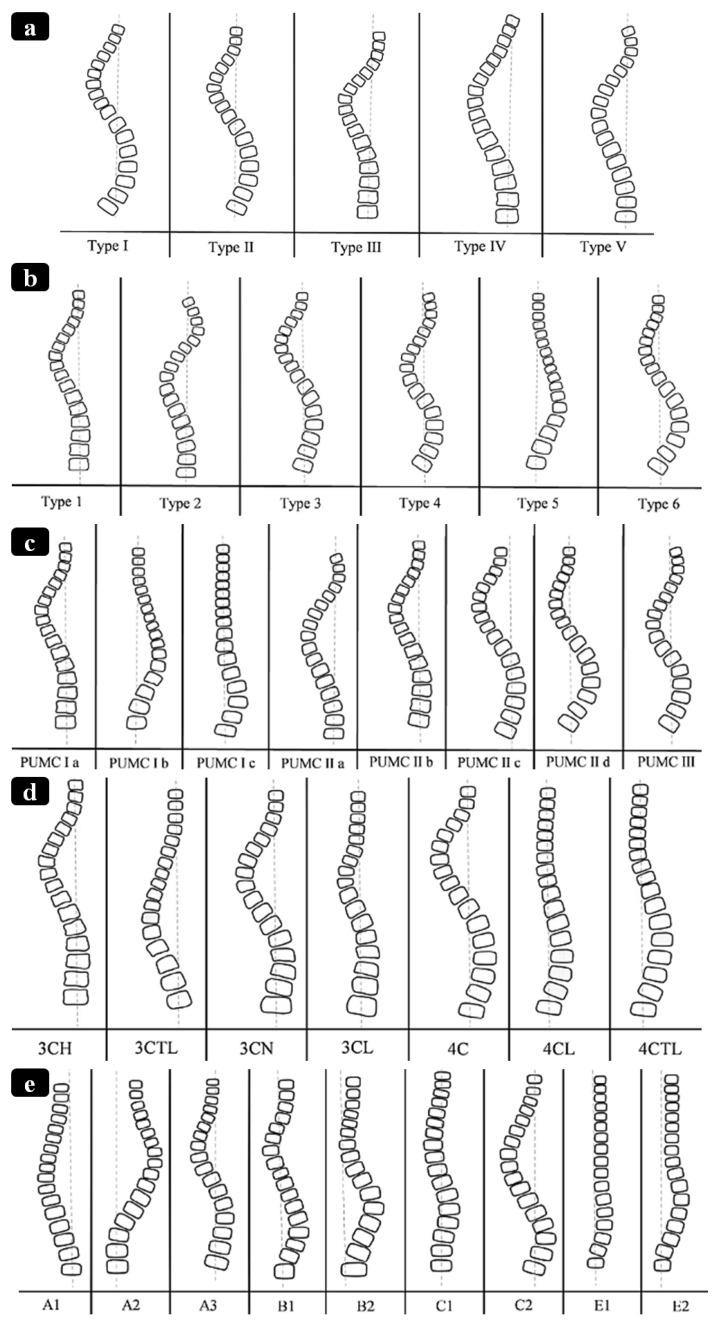
Schematic diagram of curve types [189]: (**a**) King classification, (**b**) Lenke classification, (**c**) PUMC classification, (**d**) augmented Lehnert–Schroth (ALS) classification, (**e**) Rigo classification.

Classification systems for adolescent idiopathic scoliosis (AIS) are fundamental for both clinical assessment and treatment planning, as they provide a standardized framework to describe curve morphology, guide surgical strategy, and evaluate outcomes. Traditional two-dimensional classification systems, such as the King, Lenke, and Lehnert-Schroth classifications, have been widely used for decades, as shown in Figure 9 [189]. These systems categorize spinal deformities based primarily on coronal plane curvature patterns observed in radiographs, emphasizing parameters such as the number of structural curves, apical vertebral rotation, and curve flexibility.

For the design of graded NiTi-SMA rods, these classification-derived stress maps can be directly translated into material and geometric gradients along the implant. In this approach, the transformation behavior (governed by martensitic start and austenitic finish temperatures, M_s_ and A_f_) and elastic modulus can be spatially varied to generate localized actuation responses that correspond to the required corrective forces.

Unlike traditional uniform rods, graded SMA rods do not necessarily need to follow a monotonic transformation temperature gradient (e.g., from lower to higher A_f_ along the length). AM enables spatial control of composition, porosity, and microstructure, allowing the designer to strategically position regions of higher or lower transformation temperatures wherever greater or lesser actuation forces are desired. For example, a higher A_f_ region can be located at the apical section to exert stronger corrective torque, while lower A_f_ regions at the proximal or distal ends maintain compliance and reduce stress concentrations at the bone implant interfaces.

**Integration into Surgical Practice**: The proposed design-to-implant framework for patient-specific, FG NiTi-SMA rods is intentionally structured to integrate with established scoliosis surgical workflows without increasing intraoperative complexity or procedural rigidity. Functional grading of stiffness, transformation temperature, and geometry is determined during routine preoperative planning using patient imaging and offline design tools, within timelines comparable to current elective scoliosis procedures. The rods are delivered to the operating room in a mechanically stable state compatible with standard posterior instrumentation, preserving conventional handling, fixation, and contingency options, including rod exchange if needed. Importantly, full corrective actuation is not assumed to occur intraoperatively; instead, correction can be deferred, staged, or modulated postoperatively based on clinical judgment. This preserves intraoperative flexibility and enables the management of unexpected findings while also enabling longitudinal, physician-controlled adjustment through superelastic compliance and conservative activation strategies during follow-up.

**Postoperative Monitoring and Intervention Strategy:** In conventional scoliosis instrumentation, postoperative control is limited: posterior fusion fixes correction intraoperatively and can only be altered through revision surgery, while vertebral body tethering relies on growth modulation with limited direct adjustability. In contrast, FG NiTi-SMA rods enable a supervised, longitudinal control paradigm in which corrective behavior can be monitored and modulated over time. Postoperative assessment relies on standard clinical and radiographic follow-up to detect excessive correction, imbalance, or atypical mechanical response. If deviations are observed, further activation may be paused, staged protocols adjusted, or correction accommodated through the intrinsic superelastic compliance of the rod, offering intermediate intervention options prior to surgical revision. Importantly, FG NiTi-SMA rods remain fully compatible with existing spinal instrumentation systems, preserving established revision pathways. Thus, the proposed approach augments, rather than replaces, current safety practices by shifting correction from a single irreversible intraoperative event to a monitored, incremental process under surgeon oversight.

**Figure 10:**
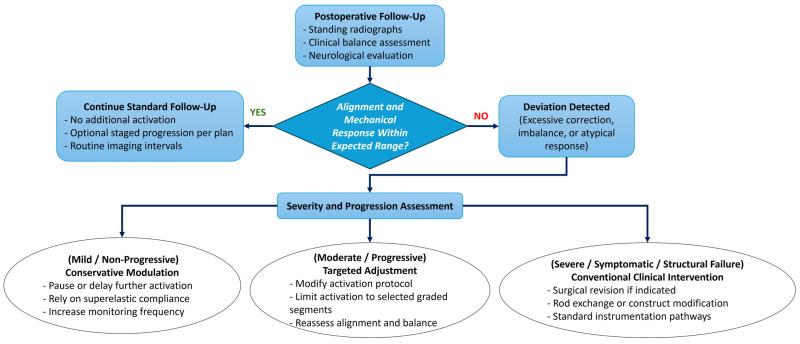
Conceptual postoperative monitoring and intervention framework for FG NiTi-SMA rods.

Figure 10 illustrates a conservative, clinician-led decision pathway for postoperative assessment, modulation, and escalation, emphasizing compatibility with standard clinical follow-up and revision strategies, shown for conceptual purposes only.

**Clinical Risk and Preclinical Validation Considerations:** From a clinical risk perspective, the most critical failure modes for FG NiTi-SMA rods include thermomechanical fatigue and functional degradation, corrosion and nickel ion release, junctional and construct-level biomechanical complications, and unintended or excessive actuation. FG NiTi rods are exposed to long-term cyclic bending, torsion, and axial loading superimposed on stress- and temperature-driven phase transformations, raising concerns related to both structural fatigue fracture and functional fatigue, such as transformation-temperature drift or reduced recoverable strain. Corrosion and ion release must be evaluated under coupled mechanical and chemical loading conditions relevant to spinal implantation, given the extended service life and high mechanical demands of scoliosis constructs. Junctional problems remain a potential risk despite graded stiffness intended to smooth load transfer, particularly if gradients or corrective moments are improperly tuned. In addition, SMA-specific risks related to unintended activation underscore the need for conservative transformation-temperature windows and surgeon-directed modulation. Accordingly, clinical translation should be preceded by rigorous preclinical validation, including multiaxial thermomechanical fatigue testing, corrosion and ion-release assessment, construct-level spine simulator testing, and computational evaluation of worst-case loading scenarios, followed by phased clinical introduction with enhanced surveillance. This framework does not eliminate surgical risk but aims to shift risk management toward earlier detection and lower-risk intervention while preserving established revision pathways.

### 7.1. Illustrative End-to-End Workflow (From Imaging to Graded Rod)

This section presents an author-proposed conceptual framework intended to synthesize existing knowledge and define development objectives; it does not represent a validated design, optimization, or clinical workflow. To this point, this work proposes an integrated pipeline that converts patient anatomy and curve classification into an FG NiTi-SMA rod with spatially tuned TTs, stiffness, and geometry:

• Patient data and curve typing: Acquire standing PA/LAT radiographs (and low-dose CT if indicated); classify curves (Lenke/Rigo/ALS) to identify apex levels, structural segments, and flexibility indices that govern where higher corrective moments versus compliance are needed.
• Target force/moment field: Using instrumented data, inverse FE from pre-/post-rod shapes, or analytical/multibody models; compute a level-wise target bending-moment/torque distribution that achieves the planned correction while respecting safe loads at screws/discs.
• Design parameterization: Discretize the rod into N segments with design variables per segment: {A_f_ (or A_s_); porosity/relative density (effective E); cross-section, I; hysteresis width, ΔT; optional surface state/texture}. A map links these to local plateau stress, stiffness, and actuation response.
• Optimization and constraints: Solve for a spatial design vector that minimizes deviation from the target moment field, subject to clinical safety (temperature limits), material (TT/plateau bounds), manufacturing (AM windows), and fatigue constraints.
• AM route and build plan: Select PBF-LB/BJAM/DED per required gradient granularity and contamination risk; generate a process map (composition/path/heat treatment) to realize the solved grading profile.
• Verification and QA: Perform segment-wise DSC, micro-CT, EBSD, indentation, and bench bending/torsion in PBS; iterate design ↔ fabrication until acceptance.
• Surgical plan and activation: Use cold shaping in martensite for contouring and then safe, controlled activation policies (SE-dominant vs. thermo-assisted) with documented torque corridors.

An axial grading mask is defined along the rod to specify regions that should be stiff/low A_f_ (higher bending moment) versus compliant/high A_f_ (lower bending moment). Vertebral levels at the deformity apex ± 1 are assigned greater moment capacity (achieved via slightly reduced A_f_ and/or increased second moment of area (I)), whereas proximal and distal zones are biased toward compliance to smooth junctional transitions. When sagittal and coronal objectives conflict, bi-objective weighting favors restoration of thoracic kyphosis around the main thoracic apex while preserving the required coronal correction. This mask initializes the optimizer and preserves consistency with classification-derived maps. Inputs are standardized as a level-wise target moment vector, $M^{*}=\{M_{1},\ldots ,M_{n}\}$, with uncertainty bounds (±Δ), accompanied by screw/disc safety constraints and an activation policy: (a) superelastic-dominant (A_f_ ≤ body temperature) for continuous, motion-responsive correction or (b) thermo-assisted staged activation (A_f_ staggered within clinically safe limits) to deliver controlled increments of correction. The force-estimation approaches below supply this vector and enforce the corresponding constraints.

Various approaches have been proposed in the literature to estimate the corrective forces exerted on vertebrae during scoliosis surgery, reflecting the complexity of the three-dimensional spinal deformation and the interaction between the implant system and the vertebral column. Early experimental techniques relied on instrumented pedicle screws or strain gauges mounted on rods to directly record the load transfer during correction maneuvers [190]. More recently, computational and imaging-based methods have gained attention. Finite element (FE)-based inverse analysis has been widely used, in which the pre- and postoperative geometries of spinal rods are reconstructed, and the corrective forces are iteratively determined so that the simulated rod deformation matches the actual postoperative shape [191]. Some studies have further incorporated patient-specific CT or radiographic data to improve anatomical accuracy, enabling calculation of localized 3D force vectors at each fixation level [123]. Complementary approaches include analytical beam-bending models and multi-body dynamic simulations that account for rod stiffness, screw positions, and vertebral coupling. Together, these techniques provide a quantitative framework to assess force distribution along the spinal construct, offering insights for optimizing rod design, fixation strategy, and personalized surgical planning in scoliosis correction.

For each segment, i, the design vector is defined as

$$x_{i}=\{Af_{i},\;\;\phi_{i}\;\;(\mathrm{porosity}/\mathrm{relative}\;\mathrm{density}),\;I_{i},\;\;\Delta T_{i},\;S_{i}\;\;(\mathrm{surface}/\mathrm{finish}),\;\;\mathrm{optional}\;\mathrm{texture}\;\mathrm{target}\}.$$

The weighted objectives are (i) moment tracking as described in Equation (1),

(1) $$\sum_{i}w_{i}\;[M_{i}(x_{i})-M_{i}^{*}{]}^{2},$$

(ii) minimization of adjacent-level stress discontinuities and screw-head contact pressures, and (iii) maximization of fatigue margin under the expected bending/torsion spectra in PBS. Hard constraints include clinical thermal safety limits; material functional windows (plateau stress, hysteresis, and recoverable strain); additive-manufacturing manufacturability bounds (composition, porosity, and geometry limits by process route); geometric compatibility with screw systems; and compliance with a constant-life fatigue curve. A surrogate-assisted multi-objective optimization is employed, and finalist designs are validated via finite element analysis using the calibrated SMA constitutive law. To predict segment-wise moment curvature plateaus and recovery stresses inside the instrumented spine, we employ a temperature-dependent SMA law consistent with Tanaka-type evolution: total strain $\varepsilon =\varepsilon^{e}+\varepsilon^{tr}$ with martensite fraction, ξ, evolving under stress–temperature thresholds (separate forward/reverse surfaces) that encode hysteresis. This provides moment curvature loops across relevant temperatures for SE-dominant and thermo-assisted activation policies. Segment-wise DSC fixes M_s_/M_f_/A_s_/A_f_; tensile/bending in PBS identifies transformation strain, Λ, and plateau stresses; indentation/ultrasound map, E, versus porosity/texture; EBSD bounds anisotropy. Accept if A_f_ within ±1.5 °C, plateau stress ± 7%, E ± 10%, and loop area (damping) ± 10% of the target. These tolerances propagate into the optimizer’s constraints and QA gates. It is worth mentioning that representative A_f_ tolerances (±1–2 °C) and stress plateau ranges (±5–10%), drawn from the reported literature trends, are used here solely to illustrate sensitivity and trade-offs rather than to prescribe validated clinical thresholds.

In this study, the Tanaka model was implemented in MATLAB R2024a to reproduce the superelastic response of the shape memory alloy at selected temperatures (e.g., $T_{a}$). The forward (A → M) and reverse (M → A) transformation branches were simulated independently using temperature-dependent material parameters. Although this phenomenological formulation does not explicitly capture partial transformations or rate effects, it accurately reproduces the essential pseudoelastic hysteresis observed in the stress–strain response under isothermal loading.

The total strain, ε, is expressed in Equation (2) as the sum of elastic and transformation contributions:

(2) $$\varepsilon =\frac{\sigma }{E}+\varepsilon_{t}$$

where σ is the applied stress, E is Young’s modulus, and $\varepsilon_{t}$ is the transformation strain associated with the phase change between austenite and martensite. The transformation strain is modeled in Equation (3) as a smooth function of stress and temperature using a hyperbolic tangent relationship:

(3) $$\varepsilon_{t}=\frac{\varepsilon_{L}}{2}[tanh(\alpha (\sigma -\sigma_{0}))+1]$$

where $\varepsilon_{L}$ is the maximum transformation strain (typically 3–6% for NiTi), α is a stress-sensitivity parameter that controls the sharpness of the transformation, and $\sigma_{0}$ represents the center stress of the transformation (the stress at which 50% transformation occurs). The parameters α and $\sigma_{0}$ are temperature-dependent and differ between the forward (austenite → martensite) and reverse (martensite → austenite) transformations. They are expressed in Equations (4)–(7):

(4) $$\alpha_{AM}=\frac{k}{C_{f}(M_{s}-M_{f})}$$

(5) $$\alpha_{MA}=\frac{k}{C_{m}(A_{f}-A_{s})}$$

(6) $$\sigma_{0,AM}=C_{f}(T-M_{P})$$

(7) $$\sigma_{0,MA}=C_{m}(T-A_{P})$$

where $C_{f}$ and $C_{m}$ are the Clausius–Clapeyron slopes for the forward and reverse transformations (MPa/K); $M_{s},M_{f},A_{s},A_{f}$ are the characteristic transformation temperatures (martensite start/finish, austenite start/finish); $M_{P}\;$ and $A_{P}$ are reference temperatures that define the midpoint of transformation; and k (≈2.9444) is an empirical constant that ensures 95% of the transformation occurs across the transformation window. Finally, combining both the elastic and transformational terms gives Equation (8), which produces a continuous stress–strain curve exhibiting the forward and reverse transformation branches typical of SMA superelasticity.

(8) $$\varepsilon =\frac{\varepsilon_{L}}{2}tanh(\alpha (\sigma -\sigma_{0}))+\frac{\sigma }{E}+\frac{\varepsilon_{L}}{2}$$

**Figure 11:**
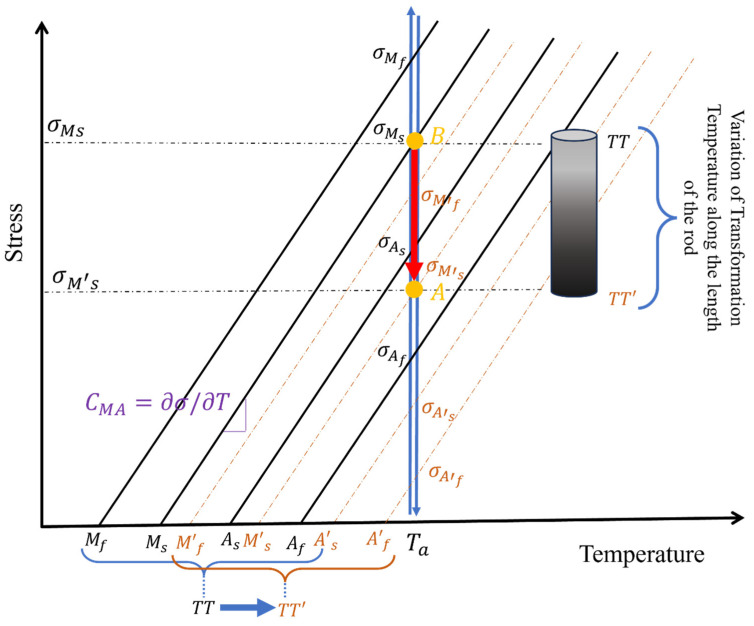
Schematic illustrating the concept of a transformation-temperature (TT) distribution along a functionally graded NiTi rod, designed based on the Clausius–Clapeyron relationship to correspond with different vertebral segments of a scoliotic spine. As the TT is increased toward one end of the rod (TT → TT′), the extent of stress-induced phase transformation is progressively reduced along the rod (B → A).

The initial objective here is to demonstrate the model’s ability to predict stress–strain behavior at specific temperatures. Subsequently, the influence of transformation temperature on the stress-induced phase transformation was analyzed, revealing the sensitivity of transformation stresses to thermal conditions. This relationship was further examined through the Clausius–Clapeyron law (illustrated in Figure 11), which quantifies the linear dependence between transformation stress and temperature. By leveraging this thermomechanical coupling, the distribution of required corrective stresses along different vertebral levels can be related to spatial variations in transformation temperature. This principle underpins the design of functionally graded NiTi rods, wherein localized adjustments of transformation temperatures allow the rod to deliver patient-specific corrective forces and tailored mechanical responses along the spine.

To ensure that stress-induced phase transformation remains manageable (depending on the required force applied to each vertebra), the initial stress for forward transformation at a given ambient temperature TaT_aTa is assumed to follow Equation (6). Later, by increasing the transformation temperature, this equation can be rewritten as Equation (9):

(9) $$\sigma_{M^{\prime }s}\left(T_{a}\right)=C_{M}(T_{b}-M_{p^{\prime }})$$

By dividing these two equations, we have Equation (10):

(10) $$\frac{\sigma_{Ms}\left(T_{a}\right)}{\sigma_{M^{\prime }s}\left(T_{a}\right)}=\frac{T_{a}-M_{p}}{T_{a}-M_{p^{\prime }}}$$

By expressing the governing equation as a relation between the martensitic peak stress corresponding to one set of transformation temperatures and that of another, we obtain

(11) $$M_{p^{\prime }}=T_{a}-\frac{\sigma_{M^{\prime }s}\left(T_{a}\right)}{\sigma_{Ms}\left(T_{a}\right)}+M_{p}.\frac{\sigma_{M^{\prime }s}\left(T_{a}\right)}{\sigma_{Ms}\left(T_{a}\right)}$$

It should be noted that the stress required at each vertebral level varies depending on the geometry and severity of the spinal curvature, which are patient-specific and adjustable during treatment planning. In certain cases, the value of $\sigma_{M^{\prime }s}(T_{a})$ may be lower, higher, or even equal to $\sigma_{Ms}(T_{a})$. Therefore, the absolute form of the expression can be applied to the last term in Equation (12):

(12) $$M_{p^{\prime }}=T_{a}+M_{p}.\left|\frac{\sigma_{M^{\prime }s}\left(T_{a}\right)}{\sigma_{Ms}\left(T_{a}\right)}-1\right|$$

In Figure 11, the first set of transformation temperatures represents the baseline condition, in which each TT value corresponds to the stress level required to achieve correction at a particular vertebra. The updated TT distribution, derived from the proposed methodology, reflects an optimized gradient where transformation temperatures are locally adjusted based on the stress requirements of the upper and lower vertebrae. In regions where higher corrective forces are needed (typically near the apex of curvature), a higher TT ensures that the stress-induced phase transformation occurs at elevated stress levels, thereby enhancing stiffness and corrective capacity. Conversely, in regions experiencing lower mechanical demand, a lower TT facilitates earlier transformation and compliance, allowing for smoother force distribution along the spinal construct. This graded TT profile enables the rod to deliver spatially tailored superelastic responses, ensuring biomechanical compatibility and effective three-dimensional correction of the spinal curvature.

**Figure 12:**
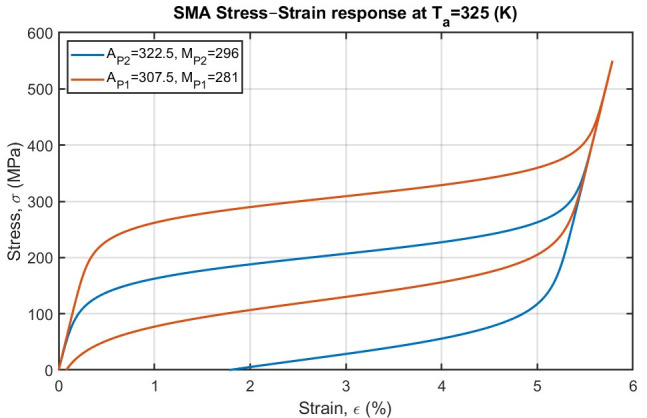
SMA superelasticity response using a distinct set of transformation temperatures at certain temperatures.

Figure 12 illustrates the simulated isothermal stress–strain response of the SMA at T = 325 K based on the Tanaka constitutive model, highlighting the fundamental mechanism underlying the proposed functionally graded structure concept for scoliosis rods. The blue curve represents the baseline material with higher transformation temperatures (A_p2_ = 322.5 K, M_p2_ = 296 K), demonstrating classical pseudoelastic behavior. Upon loading, the initial linear elastic region of austenite is followed by the forward transformation (A → M), characterized by a stress plateau associated with martensitic phase evolution. During unloading, the reverse transformation (M → A) occurs at lower stresses, forming the characteristic hysteresis loop that governs energy dissipation and recovery capability. The orange curve corresponds to a segment with lower transformation temperatures (A_p1_ = 307.5 K, M_p1_ = 281 K), which shifts the transformation stresses to higher levels at the same operating temperature. This stress shift is the key principle behind the FGS design: by spatially tailoring transformation temperatures along the rod, different segments activate transformation at different stress levels under the same global deformation. Consequently, the rod can exhibit graded stiffness and controlled load sharing, enabling more uniform strain distribution and progressive correction forces in scoliosis treatment. The material parameters employed in the simulations are summarized in Table 3. 

Building upon this foundation, the transformation temperature was systematically varied according to the proposed methodology to investigate its influence on stress-induced phase transformation. The Clausius–Clapeyron relationship was employed to correlate transformation stress with temperature, illustrating that an increase in transformation temperature shifts the transformation stress upward, whereas a decrease lowers the stress required to initiate phase transformation. This thermomechanical coupling demonstrates that localized adjustment of TT directly governs the mechanical response and actuation force of the SMA at each segment of the structure.

In the context of spinal correction, the first set of transformation temperatures in the simulation represents the condition corresponding to a specific vertebral level, reflecting the stress required to achieve correction at that point. The second set of transformation temperatures, introduced through the updated methodology, corresponds to the adjacent vertebrae, either upper or lower, where the mechanical demand differs. By tuning TT spatially along the rod, the stress–strain response can be modulated such that regions requiring higher corrective force exhibit elevated transformation temperatures, while regions demanding lower forces operate at reduced transformation temperatures.

### 7.2. Visualization Pipeline for Classification-Informed Graded Rod Design

We created a compact visualization pipeline to (a) depict a representative AIS curve pattern and clinical landmarks, (b) map a classification-informed distribution of corrective demand along the spine, and (c) translate that demand into a spatially graded transformation-temperature (*A*f) profile for a NiTi-SMA rod. A stylized coronal projection of the spine is generated as a smooth centerline parameterized by arc coordinate, s∈[0,1]:

- Lateral deviation: x(s)=0.15sin[2.4π(s−0.15)];
- Cranio-caudal coordinate: y(s)=s.

The apical vertebra is defined at the index of maximum ∣x(s)∣. Neutral vertebrae are selected near the inferior and superior ends (8% and 92% of the arc). Discs placed along (x,y) emulate vertebrae for visual context. To emulate how curve classification informs biomechanics, we assign a relative corrective-demand profile peaking at the apex:

(13) $$\tilde{\sigma }\left(s\right)\;\;=\;\alpha +\beta exp\left(-\frac{(s-s_{apex}{)}^{2}}{\lambda^{2}}\right),\;\;\;\;\mathrm{normalized}\;\mathrm{to}\;[0,1],$$

where α=0.25, β=0.75, and λ=0.12. This captures the clinical expectation that higher corrective forces are required around the apex and taper toward neutral vertebrae. The profile is rendered as a color-coded curve and is also shown as an inset plot (demand vs. arc position).

We partition the spine into low-, intermediate-, and high-demand zones using thresholds $\tilde{\sigma }<0.35$, $0.35\leq \tilde{\sigma }<0.7$, and $\tilde{\sigma }\geq 0.7$. Zone brackets are offset from the curve to avoid occlusion.

The functionally graded rod is drawn as a colored “ribbon” (constant visual width) offset laterally from the spine. The local transformation temperature is defined as a non-monotonic field:

(14) $$A_{f}(s)\;\;=\;\;A_{f,0}\;\;+\;\;A_{f,peak}exp(-\frac{(s-s_{apex}{)}^{2}}{\lambda_{A}^{2}})\;\;+\;\;\Delta A_{f}exp(-\frac{(s-s_{1}{)}^{2}}{\lambda_{1}^{2}}),$$

where $A_{f,0}={25\;}^{\circ }C$, $A_{f,peak}={10\;}^{\circ }C$, and $\lambda_{A}=0.14$, and in an optional shoulder, $\Delta A_{f}={2\;}^{\circ }C$ near $s_{1}=0.25$ and $\lambda_{1}=0.05$. The field is *min–max-*normalized to [0,1] and color-mapped so that warmer colors align with segments designed to produce greater actuation force (higher A_f_ relative to operating temperature). An inset shows $A_{f}$ versus arc position for traceability. The non-monotonic A_f_ model demonstrates that, with AM, high-A_f_ hot spots can be positioned at clinically relevant locations (e.g., apex) rather than enforcing a simple distal-to-proximal gradient.

**Figure 13:**
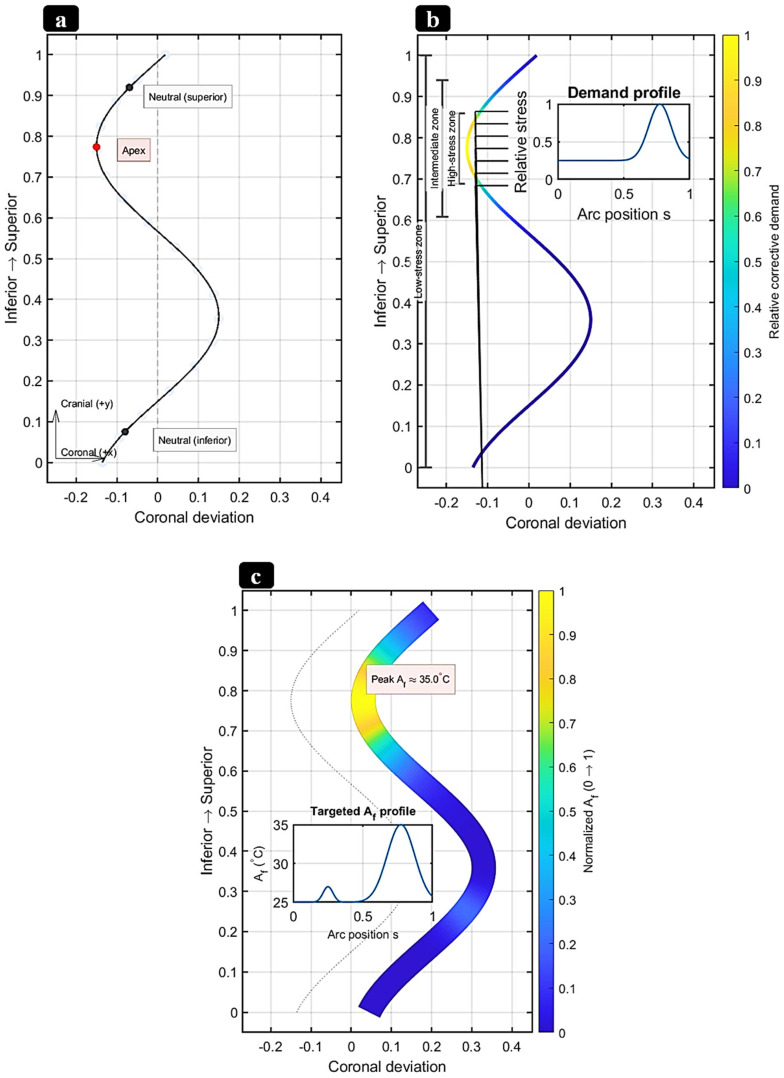
Classification-informed visualization and translation to graded NiTi-SMA rod design. (**a**) Stylized AIS coronal curve with neutral and apical vertebrae highlighted. (**b**) Normalized corrective-demand field, $\tilde{\sigma }(s)$, derived from classification logic peaks at the apex and defines low-/intermediate-/high-demand zones for design partitioning; arrows indicate the expected corrective direction. (**c**) A non-monotonic transformation-temperature profile, $A_{f}\left(s\right)$, is mapped onto a functionally graded rod, illustrating placement of higher A_f_ (greater actuation capacity) near the apex and more compliant segments near neutral levels. Insets show the scalar profiles for $\tilde{\sigma }(s)$ and $A_{f}\left(s\right)$ along the arc coordinate.

Figure 13a demonstrates a stylized AIS coronal curve (Lenke Type 1 exemplar), which highlights inferior/superior neutral vertebrae and the apical vertebra. Vertebral markers and a dashed midline provide spatial context, while small axis glyphs indicate coronal and cranial directions. Figure 13 establishes the geometric template used for subsequent biomechanical mapping. Figure 13b shows the normalized corrective-demand field $\tilde{\sigma }(s)$, which peaks at the apex and decays toward neutral vertebrae. Low-/intermediate-/high-demand regions are bracketed alongside the spine to show partitioning logic for design. Short arrows on the concave side illustrate the expected direction of corrective force. The inset provides the scalar demand profile, $\tilde{\sigma }(s)\in [0,1]$, over the arc coordinate. Figure 13c indicates that a rod ribbon, positioned parallel to the spine, is color-coded by the normalized A_f_ field, $A_{f}(s)$. The example uses a non-monotonic distribution with a peak near the apex and a small shoulder proximally, illustrating the feasibility of region-specific transformation temperatures (and, therefore, region-specific actuation capacity). The inset shows the continuous A_f_ profile, facilitating direct comparison to the demand field in Figure 13b.

## 8. Conclusions

This review consolidates the scientific and translational basis for patient-specific, functionally graded NiTi-SMA rods as a new class of scoliosis implants that can deliver targeted, time-staged correction while preserving segmental motion. On the clinical side, equipoise between fusion and non-fusion strategies remains, with comparative evidence underscoring durable correction but stiffness-related sequelae for fusion and motion preservation but mechanical uncertainty for tethering. These unresolved trade-offs motivate implants that actively modulate force and gradually adapt to growth and remodeling, capabilities intrinsic to NiTi when architected and graded appropriately.

• **Materials/biomechanics synthesis:** NiTi’s tunable martensitic transformation, near-constant plateau response, and hysteretic damping provide the physical levers for semi-active correction. When these levers are spatially graded (through composition, microstructure, and geometry), the rod can concentrate actuation near the apex and soften transitions toward neutral levels. This distributes loads more physiologically, preserves motion, and mitigates adjacent-segment stress compared with rigid constructions, aligning mechanical function with anatomic demand.
• **AM as the enabler of grading:** Among metal AM platforms, PBF-LB and DED provide the parameter space to encode gradients (energy density, scan strategy, and multi-feed alloying/thermal histories), while BJAM offers sinter-based pathways with distinct contamination risks. We linked AM choices to transformation behavior and fatigue via process–structure–property relations and highlighted mitigation/QA for Ni volatilization, O/C uptake, residual stress, and heterogeneity (parameter windows, strict atmospheres, Ni-rich feedstocks, gradient anneals, HIP/aging, and surface finishing). These controls are essential to achieve segment-wise A_f_ targets and stiffness without compromising corrosion/fatigue, and they anchor a regulatory-ready characterization plan.
• **Classification-informed design:** This work formalized a grading mask that maps curve classification to a level-wise target moment field and then to segmental design variables (A_f_, effective modulus, and cross-section). Figure 12 illustrates this pipeline: a demand map peaking at the apex is translated into a non-monotonic A_f_ profile and stiffness distribution along the rod, enabling staged, region-specific actuation with smoothed junctional transitions. This closes the loop between clinical semantics and manufacturing.
• **Constitutive modeling for predictability:** A Tanaka-type SMA law (capturing temperature-dependent hysteresis and martensite fraction evolution) provides the forward map from local design variables to moment curvature loops under physiologic temperature and loading, enabling FE-backed, uncertainty-aware optimization (e.g., tracking a target moment vector subject to screw/disc safety constraints). Within this review, modeling is positioned as the bridge from materials/process choices to construct-level performance, ensuring that segment-wise goals (plateau level, loop area/damping, and stiffness) are met before clinical translation.
• **Verification, QA, and standards:** Because small chemistry or microstructure shifts produce large TT changes, segment-wise verification is non-optional: DSC mapping of TTs, micro-CT porosity maps, EBSD/indentation for texture/modulus, and bench bending/torsion in physiologic media across curvature bands. Documentation should trace AM parameters → post-treatments → local functional properties. Alignment with recognized materials/AM standards (e.g., ASTM for NiTi and AM metals) strengthens regulatory submissions and ensures reproducibility across builds and clinics.
• **Translational implications:** With a validated grading pipeline and QA regimen, FG NiTi rods can offer patient-specific, motion-respecting correction that reduces abrupt force peaks, shares load across screws, and potentially lowers revision risk. The paradigm is not a drop-in replacement for fusion or tethering; rather, it is a complementary option for anatomies where controlled, staged correction is advantageous, and long-term compatibility with growth and motion is paramount.
• **Roadmap:** Priority research includes (i) prospective calibration between classification parameters (Cobb magnitude, apical rotation, and flexibility indices) and design metrics (A_f_ profile, stiffness gradient, and cross-sectional inertia); (ii) AM process maps that couple in situ monitoring with post-build functional metrology to guarantee graded targets; (iii) constitutive/FE digital twins for pre-operative planning and intra-operative decision support; and (iv) fatigue/corrosion protocols under multiaxial, saline environments that reflect graded cycling, with acceptance bands tied to clinical safety margins. Establishing these quantitative links will enable predictive, auditable design of next-generation FG NiTi-SMA rods.

In sum, converging evidence from SMA science, AM engineering, and spinal biomechanics supports classification-informed, AM-graded NiTi rods as a credible pathway to durable, adjustable scoliosis correction with improved postoperative biomechanical compatibility. The design, verification, and regulatory playbooks articulated here provide a translational scaffold for moving from concept to reproducible, patient-specific implants.

## Figures and Tables

**Table 1:** Comparative mechanical and material properties of biomaterials used in spinal rod implants.

Property	Ti-6Al-4V (Titanium Alloy)	316L SS	Co-Cr-Mo Alloy	NiTi SMA
**Young’s Modulus** (GPa)	110 GPa (closer to bone’s modulus than steel/CoCr) [84,85]	195–210 GPa (high stiffness vs. bone) [85]	240 GPa (very stiff; highest of these) [86]	30–75 GPa (phase-dependent; low modulus reduces stress shielding) [87]
**Yield Strength** (0.2% offset)	≥795 MPa (annealed implant-grade spec) (0.7% elastic strain before yield, calc. from E) [85,88]	310 MPa (annealed, 0% cold-work) (high-strength variants up to 758 MPa when heavily cold-worked; 0.5% elastic strain) [89]	552–758 MPa (annealed range) (can exceed 750 MPa with work-hardening; 0.2–0.3% elastic strain, from 552 MPa/241 GPa) [86]	195–690 MPa (Austenite phase; superelastic plateau range) (Martensitic phase 70–140 MPa yield; pseudoelastic plateau behavior instead of distinct yield) [90]
**Ultimate Tensile Strength**	860–950 MPa (typical UTS for annealed Grade 5/23 Ti alloy) [88]	627 MPa (annealed) (can reach 850 MPa with cold work) [90]	1030–1170 MPa (annealed) (up to 1300 MPa if hardened) [86]	895 MPa (annealed superelastic NiTi wire) (up to 1100–1300 MPa in cold-drawn condition) [90]
**Fatigue Endurance Limit**	500–600 MPa at 10^7^ cycles (high-cycle in vitro fatigue limit for smooth Ti-6Al-4V). (Rods in vivo can fail if fusion is incomplete.) [91]	300 MPa at 10^7^ cycles (estimated endurance limit for polished 316L). (Lowest fatigue resistance of these; not used in modern rods.) [92]	≥600 MPa at 10^7^ cycles (estimated; CoCr has 20–30% higher fatigue strength than Ti). (Demonstrates superior rod fatigue life vs. Ti/SS.) [93]	Can endure > 10^7^–10^8^ cycles under appropriate strain (superelastic in vitro tests show NiTi surviving 10^8^–10^9^ cycles at safe strain amplitudes). In vivo, no rod fractures were seen in 10-year series [94].
**Elastic Strain Range** (%)	0.7–1.0% elastic strain (to yield) significantly more than steel but far less than NiTi (helps avoid stress shielding to some degree).	0.2–0.5% elastic strain (exceedingly small elastic range; will plastically deform beyond 0.5%).	0.25% (approx. elastic strain to yield, due to high stiffness; limits flexibility under load—highest rigidity) (calculated from 0.2% YS and E).	6–8% recoverable strain (superelastic plateau), vastly greater elastic deflection than other metals. (Can flex without permanent setup to 8% strain.) [87]
**Surface Hardness**	340 HV (35 HRC) in annealed state. Titanium alloy is relatively soft; prone to fretting unless surface treated [95].	150 HV (79 HRB) in annealed state. Austenitic stainless steel is softer than Ti/CoCr; fretting corrosion produces abundant wear debris in vivo [89].	300 HV (30 HRC) annealed. Very hard surface; highest wear resistance (e.g., 100× better wear vs. Ti in one study) [89].	220–230 HV (annealed). It can increase with cold work. NiTi’s hardness is moderate; its wear resistance is excellent despite mid-level hardness (due to superelasticity) [96].
**MRI Artifact Profile**	**Minimal artifact:** Titanium is MR-compatible with minimal distortion, implants are highly radiolucent, aiding postop imaging [97].	**Moderate artifact:** 316L is non-ferromagnetic but causes significant MRI artifacts (much more than Ti), often obscuring detail near implants [97].	**Pronounced artifact:** CoCr is dense & has higher magnetic susceptibility, causing strong MRI artifacts. New MRI sequences can reduce (but not eliminate) this [57].	**Minimal artifact:** NiTi exhibits exceptionally low MRI interference, only minor signal loss even adjacent to implants. (NiTi stents allow lumen visualization where steel causes complete signal void.)
**Notes (In Vivo vs. In Vitro)**	**Widely used clinically:** Excellent biocompatibility and corrosion resistance in vivo. Lab values above are from ASTM specs and bench tests. In vivo, Ti rods rarely break, but fatigue failures do occur in non-union cases. Surface osseointegration is favorable, but modulus mismatch vs. bone can cause stress shielding [57].	**Historical use; now rare:** 316L was common in older implants but is phased out. In vivo, it showed inferior corrosion resistance (fretting and crevice corrosion) and generated more debris than Ti/CoCr. Modern rods favor Ti or CoCr for better MRI compatibility and longevity. (Data above from standards/bench tests.) [98].	**Used in deformity corrections:** CoCr rods are stiffer and stronger, used for multi-level fusions and scoliosis corrections. In vitro, they outperform Ti in bending fatigue. In vivo, they provide better initial deformity correction, but their high rigidity has been linked to higher adjacent-segment stress and degeneration over time. Excellent corrosion resistance in body fluids, especially when coupled with Ti screws (minimal galvanic issues).	**Emerging/dynamic use:** NiTi rods have been used in semi-rigid dynamic stabilization systems to allow slight motion. Lab tests confirm superelastic behavior and high fatigue resistance. Clinically, small series report no mechanical failures (no rod breakages) over ~10 years and reduced adjacent-level stress. Biocompatibility is high; Ni release in vivo is low and below toxic thresholds. (Most property data are from in vitro experiments and ASTM F2063 standards.)

**Table 2:** AM challenges for functionally graded NiTi-SMA scoliosis rods: mechanisms, impact on transformation/fatigue, and mitigation/QA strategies (PBF-LB, DED, BJAM).

AM Challenge	Impact on NiTi/FG Rod Performance	Possible Mitigation or Design Strategy	Ref.
**Ni evaporation during laser melting**	Loss of Ni shifts transformation temperatures (Ms, Mf, As, Af) upward, potentially eliminating superelastic behavior at body temperature or altering SME activation thresholds	Use Ni-enriched powder feedstock to compensate for evaporation losses; optimize laser energy density and scanning strategies; apply chamber atmosphere control to minimize evaporation; include post-process heat treatments to re-stabilize transformation temperatures	[164,165]
**Oxidation and impurity uptake**	Formation of oxide or carbides (TiO_2_, TiC, Ni-oxide) and oxygen contamination can deplete Ti, lead to secondary phases, reduce functional strain recovery, and degrade fatigue life	Maintain low oxygen environment during build and powder handling; implement in situ monitoring of melt pool chemistry; apply post-fabrication deoxidation or annealing to reduce oxide layers; surface passivation treatments to control oxide scale	[166]
**Residual stress, cracking, and distortion**	Residual stress from rapid thermal cycling can cause warping, cracking, or fatigue initiation. These mechanical defects degrade rod integrity, reduce fatigue life, and may compromise transformation behavior	Use optimized scanning strategies (e.g., bidirectional scanning, adaptive rescan, contour scans) to reduce thermal gradients; incorporate intermediate stress-relief heat treatments; design support structures and build orientations to minimize distortion; monitor in situ and apply real-time mitigation	[167]
**Microstructural heterogeneity and precipitate formation**)	Formation of precipitates (e.g., Ni_4_Ti_3_), secondary phases, or phase segregation can alter transformation behavior, reduce strain recovery, and promote local mechanical or transformation mismatches, especially in graded zones.	Tailor heat treatment protocols (annealing, aging) to homogenize microstructure and dissolve or control precipitates; incorporate gradient post-processing to align with graded composition; use in situ alloying methods to smooth transitions; conduct careful DSC/EBSD characterization across graded segments	[147]
**Fatigue and transformation stability under cyclic loading**	Long-term cyclic thermomechanical loading and phase transformation can cause drift in transformation temperatures, functional degradation (reduced strain recovery), and eventual fatigue failure, especially problematic spinal rods subject to bending, torsion, and cycling.	Perform accelerated cyclic fatigue testing under representative thermal–mechanical conditions; design graded transformation profiles to limit full transformation cycle; include protective surface layers or coatings to reduce mechanical wear; embed sensor-based monitoring of transformation drift in design	[167,168]
**Complexity in graded fabrication and QA of FG zones**	Spatial variations in composition, geometry, or heat treatment make quality assurance challenging; small deviations in grading may yield large local changes in activation temperature or stiffness, potentially undermining the intended gradient design.	Use in situ monitoring (melt pool, powder feed, temperature sensors) and post-build characterization (microstructural, DSC, microhardness mapping) across the length of the rod; develop digital twins of graded builds to predict local functional response; modular segmentation approaches to simplify grading; use process-mapped grading protocols	[166]

**Table 3:** Superelastic properties of the NiTi material used as the model input data [192].

	E (GPa)	$C_{f}$ [MPa/K]	$C_{r}$ [MPa/K]	$\varepsilon_{tran}$ [%]	$M_{p}$ [K]	$A_{p}$ [K]	$M_{s}$ [K]	$M_{f}$ [K]	$A_{s}$ [K]	$A_{f}$ [K]
*NiTi*	70	7	7	5	281	307.5	291	271	295	320

## Data Availability

This is a review article and does not report new experimental data. All data discussed in this paper are derived from previously published studies, which are properly cited in the reference section. Any supporting materials or figures generated by the authors are available from the corresponding author upon reasonable request.

## Abbreviations

**Abbreviation**	**Definition**
**316L**	AISI 316L Stainless Steel
**A_f_**	Austenite Finish Temperature
**AIS**	Adolescent Idiopathic Scoliosis
**AM**	Additive Manufacturing
**A_s_**	Austenite Start Temperature
**ASTM**	American Society for Testing and Materials
**B19′**	Martensite Phase (Monoclinic B19′)
**B2**	Austenite Phase (Cubic B2)
**BJAM**	Binder Jet Additive Manufacturing
**BSE**	Backscattered Electron (Imaging Mode)
**CoCr**	Cobalt–Chromium Alloy
**CT**	Computed Tomography
**DED**	Directed Energy Deposition
**FDA**	U.S. Food and Drug Administration
**FE**	Finite Element
**FE-SEM**	Field-Emission Scanning Electron Microscopy
**FG**	Functionally Graded
**FGM**	Functionally Graded Material
**FGS**	Functionally Graded Structure(s)
**HIP**	Hot Isostatic Pressing
**HRB**	Rockwell B Hardness
**HRC**	Rockwell C Hardness
**HV**	Vickers Hardness
**IPF**	Inverse Pole Figure
**LPBF**	Laser Powder Bed Fusion (Synonym of PBF-LB)
**MCGR**	Magnetically Controlled Growing Rod
**M_f_**	Martensite Finish Temperature
**MRI**	Magnetic Resonance Imaging
**Ms**	Martensite Start Temperature
**NiTi**	Nickel–Titanium (Nitinol)
**O/N**	Oxygen/Nitrogen Content
**OWSME**	One-Way Shape Memory Effect
**PBF-LB**	Powder Bed Fusion—Laser Beam
**PEEK**	Polyether Ether Ketone
**PSF**	Posterior Spinal Fusion
**QA**	Quality Assurance
**R-phase**	Rhombohedral Intermediate Phase in NiTi
**SE**	Superelasticity
**SMA**	Shape Memory Alloy
**SME**	Shape Memory Effect
**STEM**	Scanning Transmission Electron Microscopy
**Ti-6Al-4V**	Titanium Alloy (grade Ti-6Al-4V)
**TT**	Transformation Temperatures (M_s_, M_f_, A_s_, A_f_)
**TWSME**	Two-Way Shape Memory Effect
**VBT**	Vertebral Body Tethering
**YS**	Yield Strength
